# The Contribution of GWAS Loci in Familial Dyslipidemias

**DOI:** 10.1371/journal.pgen.1006078

**Published:** 2016-05-26

**Authors:** Pietari Ripatti, Joel T. Rämö, Sanni Söderlund, Ida Surakka, Niina Matikainen, Matti Pirinen, Päivi Pajukanta, Antti-Pekka Sarin, Susan K. Service, Pirkka-Pekka Laurila, Christian Ehnholm, Veikko Salomaa, Richard K. Wilson, Aarno Palotie, Nelson B. Freimer, Marja-Riitta Taskinen, Samuli Ripatti

**Affiliations:** 1 Institute for Molecular Medicine Finland FIMM, University of Helsinki, Helsinki, Finland; 2 Program in Medical and Population Genetics, The Broad Institute of MIT and Harvard, Cambridge, Massachusetts, United States of America; 3 Psychiatric & Neurodevelopmental Genetics Unit, Department of Psychiatry, Massachusetts General Hospital, Boston, Massachusetts, United States of America; 4 Research Programs Unit, Diabetes & Obesity, University of Helsinki, and Heart and Lung Centre, Helsinki University Hospital, Helsinki, Finland; 5 Endocrinology, Abdominal Center, Helsinki University Hospital, Helsinki, Finland; 6 Department of Human Genetics, David Geffen School of Medicine at UCLA, University of California Los Angeles (UCLA), Los Angeles, California, United States of America; 7 National Institute for Health and Welfare, Helsinki, Finland; 8 Center for Neurobehavioral Genetics, Semel Institute for Neuroscience and Human Behavior, University of California, Los Angeles, California, United States of America; 9 Public Health Genomics Unit, National Institute for Health and Welfare, Helsinki, Finland; 10 Department of Medical Genetics, University of Helsinki, Helsinki, Finland; 11 McDonnell Genome Institute, Washington University School of Medicine, St. Louis, Missouri, United States of America; 12 Analytic and Translational Genetics Unit, Department of Medicine, Massachusetts General Hospital, Boston, Massachusetts, United States of America; 13 The Stanley Center for Psychiatric Research, The Broad Institute of MIT and Harvard, Cambridge, Massachusetts, United States of America; 14 Department of Neurology, Massachusetts General Hospital, Boston, Massachusetts, United States of America; 15 Department of Public Health, Clinicum, Faculty of Medicine, University of Helsinki, Helsinki, Finland; 16 Wellcome Trust Sanger Institute, Cambridge, United Kingdom; Georgia Institute of Technology, UNITED STATES

## Abstract

Familial combined hyperlipidemia (FCH) is a complex and common familial dyslipidemia characterized by elevated total cholesterol and/or triglyceride levels with over five-fold risk of coronary heart disease. The genetic architecture and contribution of rare Mendelian and common variants to FCH susceptibility is unknown. In 53 Finnish FCH families, we genotyped and imputed nine million variants in 715 family members with DNA available. We studied the enrichment of variants previously implicated with monogenic dyslipidemias and/or lipid levels in the general population by comparing allele frequencies between the FCH families and population samples. We also constructed weighted polygenic scores using 212 lipid-associated SNPs and estimated the relative contributions of Mendelian variants and polygenic scores to the risk of FCH in the families. We identified, across the whole allele frequency spectrum, an enrichment of variants known to elevate, and a deficiency of variants known to lower LDL-C and/or TG levels among both probands and affected FCH individuals. The score based on TG associated SNPs was particularly high among affected individuals compared to non-affected family members. Out of 234 affected FCH individuals across the families, seven (3%) carried Mendelian variants and 83 (35%) showed high accumulation of either known LDL-C or TG elevating variants by having either polygenic score over the 90^th^ percentile in the population. The positive predictive value of high score was much higher for affected FCH individuals than for similar sporadic cases in the population. FCH is highly polygenic, supporting the hypothesis that variants across the whole allele frequency spectrum contribute to this complex familial trait. Polygenic SNP panels improve identification of individuals affected with FCH, but their clinical utility remains to be defined.

## Author Summary

Familial combined hyperlipidemia (FCH) is a familial dyslipidemia and the most common familial risk factor for premature coronary heart disease. Its genetic architecture is poorly understood. Rare high-impact variants have been identified in some patients, but have not explained a substantial portion of the trait. FCH has previously been speculated to be a polygenic disorder, but genetic data supporting this hypothesis have so far been incomplete. We provide experimental evidence for the polygenicity and heterogeneity of FCH in a large set of affected families using comprehensive genome-wide variant data. Approximately a third of the affected FCH individuals in our sample had high polygenic burden, and only a minority carried high-impact variants identifiable by genotyping. We show that the polygenic burden of affected FCH family members is comparable to that observed in individuals with similar lipid phenotypes in the general population. Genetic variants identified in large-scale population studies can also underlie the typical phenotypes observed in complex familial diseases such as FCH. Advances in genetic diagnosis based on population samples may thus also benefit FCH families. Families without high polygenic burden are good candidates for sequencing studies to identify rare variants not observable with genotyping.

## Introduction

Familial combined hyperlipidemia (FCH), classically defined by elevations in serum total cholesterol (TC), triglycerides (TG), or both, in two or more first degree relatives, displays a prevalence of greater than 1% in Western populations [1, 2]. It is the most common familial risk factor for premature coronary heart disease (CHD), occurring in 11–14% of individuals with this condition, and raising by up to five-fold the CHD risk in first- and second-degree relatives of affected individuals [3, 4]. In clinical practice, FCH is characterized by elevations of low-density lipoprotein cholesterol (LDL-C), TG, or both [5]. The phenotype within a family shows high inter- and intraindividual variability of lipid values (TG, LDL-C, high-density lipoprotein cholesterol [HDL-C], and apolipoprotein B) and therefore the diagnosis is commonly missed. Despite attempts to identify rare high-impact variants underlying FCH, no such variant has explained a substantial proportion of the trait [6]. Also, so far the genetics of FCH has not been addressed using high-density genotyping panels [7].

Here, we present a comprehensive evaluation of the genetic background of FCH using a dense-marker genotyping panel. We hypothesized that FCH risk could derive in part from a combination of common variants associated, in population cohorts, with LDL-C and/or TG as well as uncommon variants in genes that have been implicated in Mendelian lipid syndromes. To test our hypothesis we evaluated Finnish FCH families to determine if they demonstrated an enrichment of known lipid-associated variants, compared to Finnish general population samples, and assessed whether such variants could account for differences in lipid levels and degree of aggregation of disease observed between the FCH families. Additionally, following the logic of recent studies that have demonstrated the combined impact of multiple small effect variants (polygenic scores) on risk for familial hypercholesterolemia (FH) and other lipid disorders [8–11], we measured, in these families, the relative contributions of LDL-C and TG associated polygenic scores to the lipid traits and the FCH phenotype.

## Results

**Fig 1:**
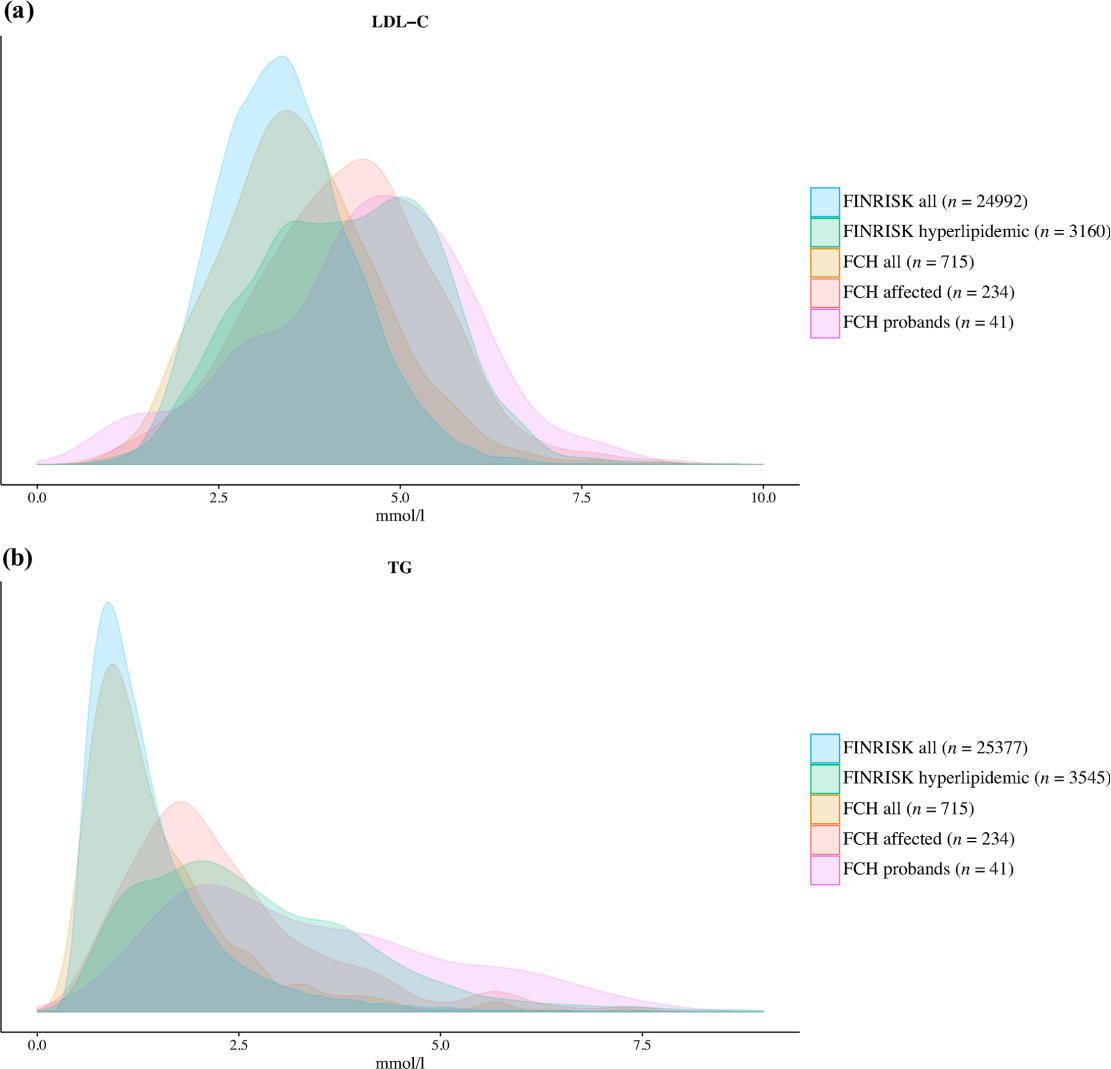
Distributions of lipid levels in subsets of the Finnish general population and the FCH samples. Distributions of (a) LDL-C and (b) TG are shown for the Finnish FINRISK population cohort (FINRISK all, blue), hyperlipidemic Finnish population samples (FINRISK hyperlipidemic, green), all FCH family members (FCH all, brown), affected family members (FCH affected, red), and proband individuals (FCH probands, purple). Hyperlipidemia in the population samples is defined as TC or TG ≥ 90^th^ age- and sex-specific population percentile, analogously with the FCH diagnostic criteria. In (b) the x-axis is cut at 9 mmol/l. FCH, familial combined hyperlipidemia; FINRISK, The National FINRISK Study.

We evaluated the contribution of lipid-level associated genetic variation to FCH in 715 genotyped individuals (234 of whom were considered affected by FCH; Table 1, S1 Table, and S1 Fig) from 53 FCH families (average family size 13 individuals). Of the 234 FCH-affected individuals, 78 (33%) were identified because of elevated (≥ 90^th^ age- and sex-specific population percentile) TC without elevated TG, 76 (32%) had elevated TG without elevated TC, and 80 (34%) had both lipids elevated. FCH probands (*n* = 48, because five probands had DNA unavailable) and other FCH affected individuals showed higher mean levels of both LDL-C (4.45 and 4.29 mmol/l, respectively) and TG (4.06 and 2.51 mmol/l, respectively) compared to all FCH family members (LDL-C and TG 3.64 and 1.60 mmol/l, respectively) and to the general Finnish population (LDL-C and TG 3.43 and 1.43 mmol/l, respectively) (Fig 1). The median LDL-C and TG values of the genotyped family members ranged from 1.08 to 4.69 mmol/l for LDL-C and from 0.70 to 4.44 mmol/l for TG demonstrating considerable variation among families (S2 Fig).

**Table 1:** Clinical and metabolic characteristics of genotyped individuals.

	FCH affected		FCH unaffected		
Characteristic	*n*	Mean ± SD	*n*	Mean ± SD	*p*-value
*n* (male/female)	234 (108/126)		481 (243/238)		0.27
Smoking, *n* (%)	75 (32)		122 (25)		0.50
Age (year)	233	42.7±14.2	477	40.6±14.8	0.08
BMI (kg/m^2^)	232	27.6±4.7	464	25.5±4.7	2.21*x*10^-6^
Waist circumference (cm)	190	93±14	370	86±14	3.80*x*10^-7^
Total cholesterol (mmol/l)	211	6.64±1.36	403	5.18±0.92	1.97*x*10^-53^
LDL-C (mmol/l)	211	4.29±1.23	403	3.30±0.89	6.64*x*10^-29^
Triglyceride (mmol/l)	211	2.51±1.85	403	1.13±0.48	3.77*x*10^-45^
HDL-C (mmol/l)	208	1.23±0.40	395	1.40±0.41	1.23*x*10^-6^
Apolipoprotein B (mg/dl)	192	126±32	378	87±22	5.74*x*10^-65^
Non-HDL-C	208	5.41±1.39	395	3.78±0.95	1.99*x*10^-62^

*P-*values were calculated using Wald test by a linear mixed model correcting for sample relatedness. FCH, familial combined hyperlipidemia.

**Fig 2:**
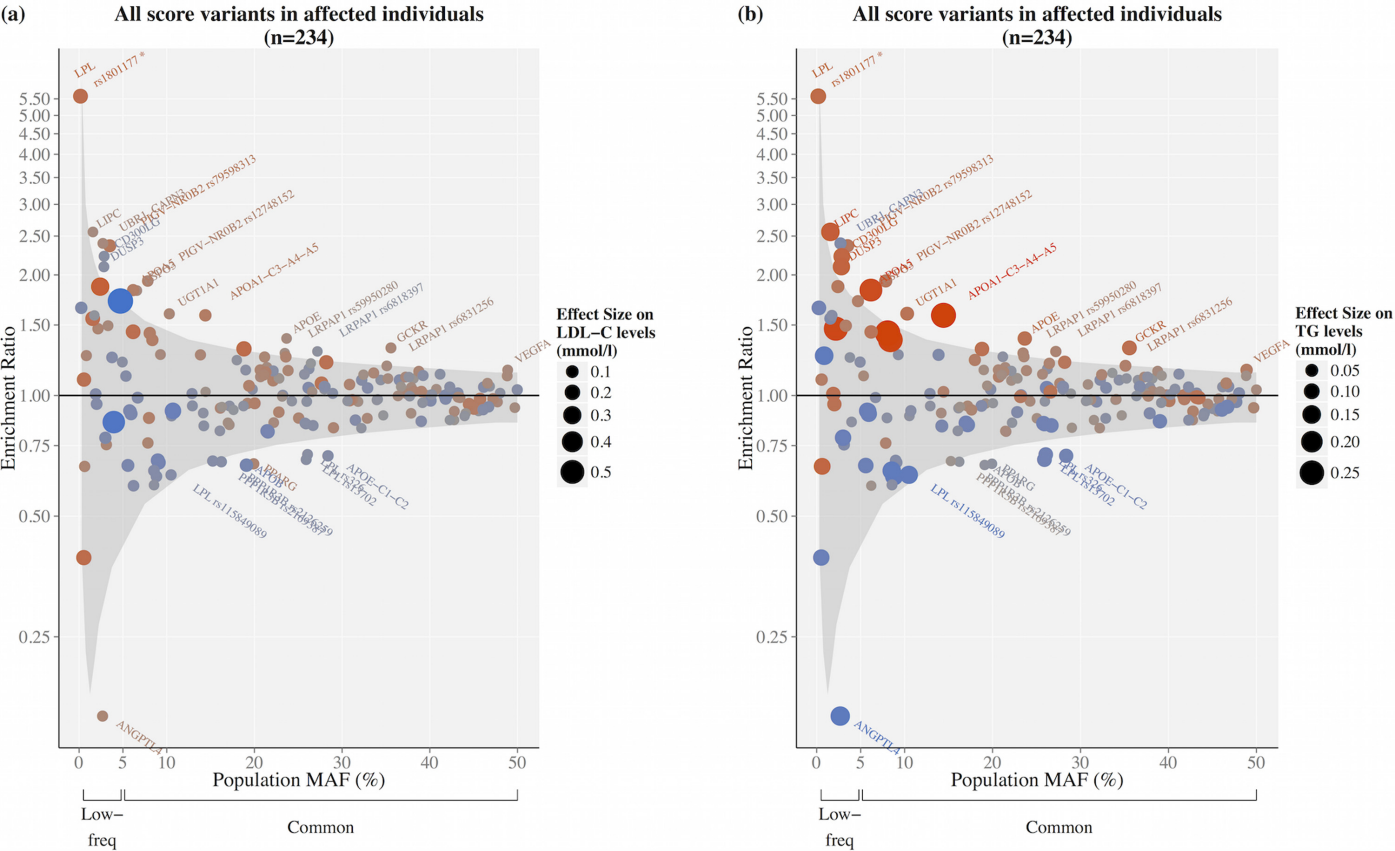
Enrichment of LDL-C or TG associated SNPs in FCH affected individuals by their frequency and effect on LDL-C and TG levels. Enrichment ratio is the ratio of the allele frequencies in the affected individuals (*n* = 234) to the allele frequencies in the Finnish FINRISK population cohort (*n* = 18,715). Only individuals without diabetes or other relevant confounders were included (S1 Text). Under the null hypothesis of no enrichment, a 95% credible interval (shaded area) was estimated by calculating the enrichment statistic (enrichment ratio) for all variants with MAF > 0.001% across the genome excluding the loci of the 212 SNPs. Variants are designated as either lipid level elevating (red) or lowering (blue) for (a) LDL-C and (b) TG based on *β* estimates from linear regression in the FINRISK samples. Point size and color intensity reflect the magnitude of the effect. Only SNPs with at least one heterozygous carrier are shown *(n* = 194). *The enrichment ratio for *LPL* rs1801177 fell within the 95% credible interval.

Compared to 18,715 random Finnish population samples, alleles that elevated LDL-C and TG levels in the population were overall enriched and alleles that lowered LDL-C and TG levels were depleted in 234 affected FCH individuals (sign test *p* = 0.0025 for LDL-C and *p* = 0.0016 for TG). We included in this analysis all SNPs with at least one affected carrier (194 of the 212 SNPs previously associated with either LDL-C or TG in population genome-wide association (GWA) studies or with monogenic dyslipidemias). In total, 60 out of 95 LDL-C elevating SNPs were more common in EUFAM than in population samples, and 57 out of 99 of LDL-C lowering SNPs were less common. Similarly, 61 out of 96 TG elevating SNPs were enriched and 57 out of 98 TG lowering SNPs were depleted (Fig 2, S3 Table). S3 Fig shows the enrichment ratios for probands and all family members compared to the population samples.

We then examined if high-impact variants implicated in monogenic dyslipidemias would be enriched and contribute to FCH. *APOA5* rs3135506, which predisposes to hypertriglyceridemia in homozygous form [12], was 1.8–fold more frequent in the affected individuals of the FCH families than in the general population (minor allele frequency [MAF] in affected FCH individuals = 0.11, MAF in population = 0.062). In all FCH family members, there were 105 heterozygotes (43 of them affected) and six homozygotes (five of them affected). Homozygotes had a mean TG level (mmol/l) of 2.61, heterozygotes 1.97 and wild type carriers 1.55 (S4 Fig). *APOE* variations have a well-established role in dyslipidemias yet incompletely defined contribution to FCH. By genotyping rs7412 and imputing rs429358 we were able to determine the phenotyped apolipoprotein E isoform with a minimum accuracy of 95% (Table 2). The *APOE* ɛ2ɛ2 haplotype that predisposes to type III hyperlipoproteinemia [13], was observed in three FCH family members (two of them affected), all with elevated cholesterol and TG concentrations in very low-density lipoprotein and intermediate-density lipoprotein fractions (Table 2). Its frequency was 0.0023 in the Finnish population, 0.0045 in all FCH families and 0.0085 among FCH affected individuals. *LIPC* rs28933094 predisposes to hepatic lipase deficiency, the cardiovascular effects of which are unclear but may be dependent on the underlying lipid phenotype [14, 15]. It is 4.8–fold more frequent in Finns compared to other Europeans and additionally 2.6–fold more frequent in affected FCH individuals compared to the Finnish population (MAF in affected FCH individuals = 0.041, MAF in Finnish population = 0.016, MAF in Non-Finnish Europeans = 0.0033). Finally, it is of special interest whether the FCH families carry any of the classical FH variants. In the Finnish population there are five major extensively documented, FH-associated *LDLR* variants [16]. As the genotyping array does not capture these, we successfully imputed one out of the five variants (FH-Pogosta), but observed no carriers. In summary, variants that are strongly linked to known forms of dyslipidemia (in *APOA5* and *APOE*), could at most explain only 7 (3.0%) of all 234 affected FCH individuals, thus being of minor importance in our FCH family sample.

**Table 2:** Apolipoprotein E phenotypes, imputed haplotypes, and the carriers’ lipid profiles.

	Apolipoprotein E phenotype (*n* = 557)					
	ε2ε2	ε2ε3	ε2ε4	ε3ε3	ε3ε4	ε4ε4
*n* (%)	3 (0.5)	54 (9.7)	16 (2.9)	272 (48.8)	191 (34.3)	21 (3.8)
Imputed APOE haplotype, *n* (%)	3 (0.5)	52 (9.3)	16 (2.9)	269 (48.3)	184 (33.0)	20 (3.6)
Accuracy of imputation* (%)	100	96.3	100	98.9	96.3	95.2
rs429358 alleles	T/T	T/T	C/T	T/T	C/T	C/C
rs7412 alleles	T/T	C/T	C/T	C/C	C/C	C/C
VLDL-C (mmol/l)	4.94±0.83	0.68±0.60	0.87±0.81	0.44±0.39	0.57±0.56	0.64±0.63
VLDL-TG (mmol/l)	3.95±1.19	1.25±0.99	2.12±3.12	0.89±0.79	1.04±1.05	1.28±1.21
LDL-C, measured (mmol/l)	1.73±0.30	2.82±0.83	3.63±0.82	3.53±0.89	3.63±0.93	3.90±1.10
LDL-TG (mmol/l)	0.29±0.08	0.26±0.13	0.32±0.18	0.26±0.09	0.29±0.13	0.28±0.11
IDL-C (mmol/l)	1.23±0.27	0.24±0.12	0.28±0.15	0.20±0.12	0.21±0.15	0.20±0.17
IDL-TG (mmol/l)	0.33±0.10	0.13±0.06	0.16±0.11	0.12±0.06	0.12±0.07	0.12±0.07

Values are presented for all FCH family members whose apolipoprotein E phenotypes were determined (*n* = 557). The *APOE* haplotype of an individual was determined only if the posterior probability of rs429358 was > 0.9 for any allele. Lipoprotein values are presented as mean ±SD. VLDL-C = very low-density lipoprotein cholesterol. VLDL-TG = very low-density lipoprotein triglyceride. IDL-C = intermediate-density lipoprotein cholesterol. IDL-TG = intermediate-density lipoprotein triglyceride. LDL-C = low-density lipoprotein cholesterol. LDL-TG = low-density lipoprotein triglyceride.

*Accuracy of imputation is the quotient of the *n* of the observed apolipoprotein E phenotype and the *n* of the accordingly imputed *APOE* haplotype.

**Fig 3:**
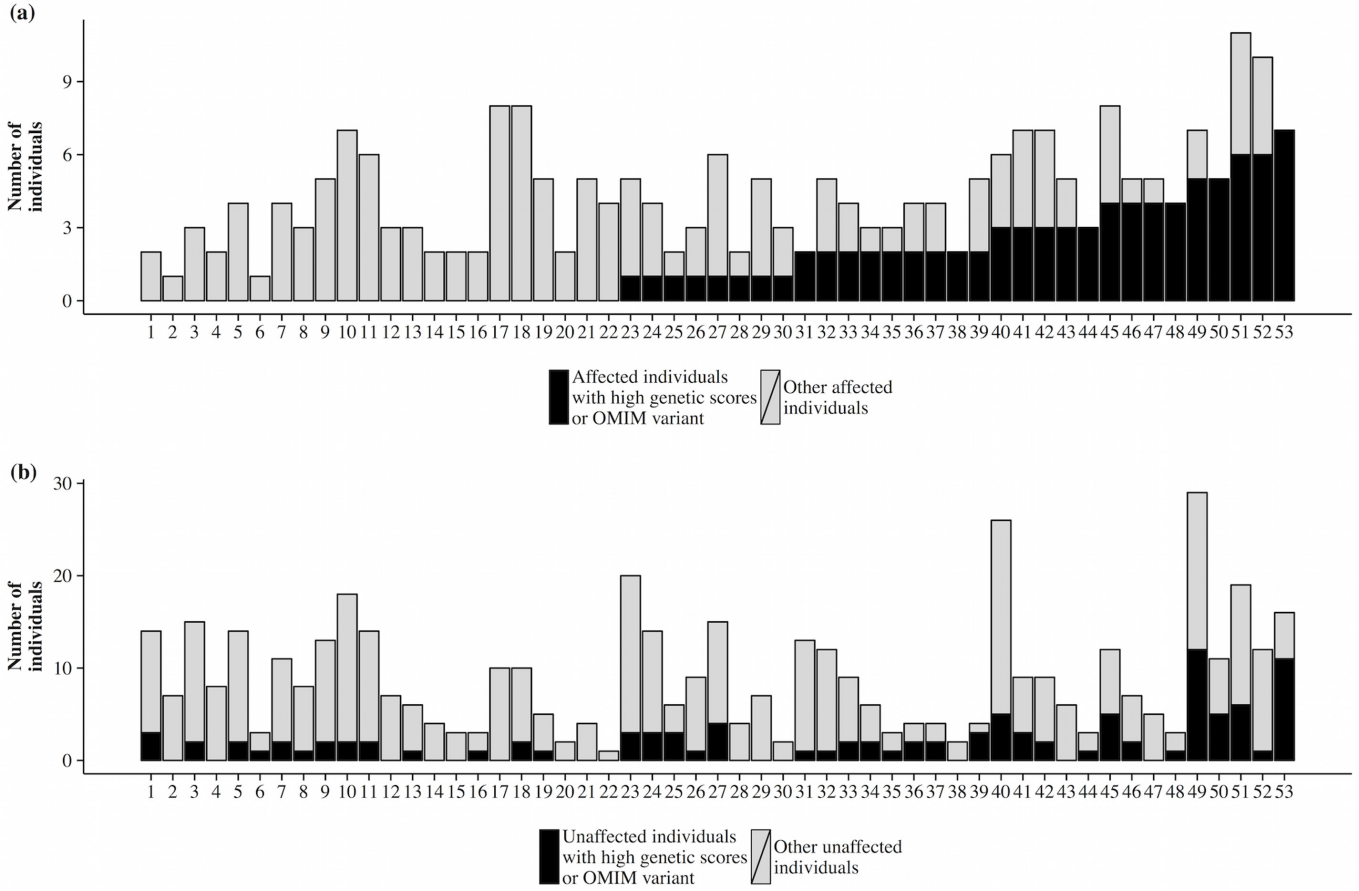
Number of affected and unaffected individuals with high polygenic lipid scores or carriers of high-impact Mendelian variants. The black shading presents the number of (a) affected, and (b) unaffected individuals with high polygenic lipid scores (LDL-C, TG, or both polygenic scores over the 90^th^ percentile in the population) or carriers of a high-impact Mendelian variant (*APOE* ɛ2ɛ2, *n* = 2; or homozygosity for *APOA5* rs3135506). The families are sorted by the number of affected individuals with high polygenic lipid scores or a high-impact Mendelian variant in (a). The grey shading presents the number of other (a) affected, and (b) unaffected individuals in the family.

We then estimated what proportion of the affected FCH individuals had a high cumulative burden of enriched LDL-C and TG elevating variants or carried high-impact variants. Out of the 234 affected FCH individuals, 83 individuals (35%) showed high accumulation of known LDL-C or TG elevating variants, having either one or both polygenic lipid scores over the 90^th^ percentile in the population (Table 3). Three additional FCH affected individuals carried Mendelian variants and did not have elevated polygenic scores. In 14 out of the 53 (25%) families, over half of the affected individuals had high polygenic scores or carried known Mendelian variants (Fig 3). In six out of the 53 (11%) families, all affected members had high polygenic scores or carried known Mendelian variants. This did not appear to be driven by differences in genetic correlation between affected individuals in the families (S5 Fig).

**Table 3:** Number of affected individuals with high polygenic lipid score or Mendelian SNPs.

	High* LDL-C score	High* TG score	Either score high	Both scores high	OMIM SNP^†^	Either score high or OMIM SNP^†^
**FCH affected (*n* = 234)**	40 (17%)	59 (25%)	83 (35%)	16 (6.8%)	7 (3.0%)	86 (37%)
**FCH probands (*n* = 48)**	2 (4.2%)	13 (27%)	13 (27%)	2 (4.2%)	2 (4.2%)	14 (29%)
**FINRISK hyperlipidemic**^**‡**^ **(*n* = 2663)**	479 (18%)	479 (18%)	839 (32%)	119 (4.5%)	29 (1.1%)	850 (32%)
**FINRISK statin users (*n* = 6300)**	934 (15%)	777 (12%)	1504 (24%)	207 (3.3%)	39 (0.62%)	1516 (24%)

OMIM, Online Mendelian Inheritance in Man; FCH, familial combined hyperlipidemia; FINRISK, The National FINRISK Study.

*Polygenic lipid score ≥ 90^th^ Finnish population percentile, calculated in the Finnish FINRISK population cohort.

^†^Carriers of SNPs that cause Mendelian dyslipidemias and are observed in the FCH families (*APOE* ɛ2ɛ2 or *APOA5* rs3135506 homozygotes).

^‡^Hyperlipidemia in the FINRISK population samples is defined as TC or TG (non-fasting) ≥ 90^th^ age- and sex-specific population percentile, analogously with the FCH diagnostic criteria.

Finally, we evaluated how well the high polygenic scores predicted FCH affectedness in the families or among hyperlipidemic individuals in the population (Table 4). Having a high polygenic score (≥ the 90^th^ population percentile) for either LDL-C or TG had a positive predictive value (PPV) of 0.45 in the FCH families and 0.23 in the general population. Negative predictive values (NPV) were 0.71 and 0.89, respectively.

**Table 4:** Positive and negative predictive values of high polygenic lipid scores.

	Prevalence	PPV of high* score				NPV of high* score			
		LDL-C	TG	Either	Both	LDL-C	TG	Either	Both
**FCH affected (*n* = 234)**	33%	0.46	0.46	0.45	0.55	0.69	0.70	0.71	0.68
**FINRISK hyperlipidemic**^†^ **(*n* = 2663)**	13%	0.23	0.23	0.23	0.30	0.88	0.88	0.89	0.87

PPV, positive predictive value; NPV, negative predictive value; FCH, familial combined hyperlipidemia; FINRISK, The National FINRISK Study.

*Polygenic lipid score ≥ 90^th^ Finnish population percentile, calculated in the Finnish FINRISK population cohort.

^†^Hyperlipidemia in the FINRISK population samples is defined as TC or TG (non-fasting) ≥ 90^th^ age- and sex-specific population percentile, analogously with the FCH diagnostic criteria.

In summary, we observed enrichment of both common and uncommon lipid-elevating variants in the FCH families. Only a minority (7 out of 234, 3%) of affected individuals were carriers of high-impact variants of Mendelian dyslipidemias. The polygenic lipid scores contribute to FCH in over one third of 234 FCH subjects in these families, with considerable heterogeneity between families. In more than half of the families we did not observe either an increased load of common variants or carriers of high-impact variants.

## Discussion

Our results demonstrate an enrichment of many known lipid-level elevating variants in FCH families. This enrichment was observed for both uncommon and common variants known to affect either LDL-C or TG levels in populations. When the known variants were combined into polygenic lipid scores for each individual, 17% and 25% of the affected individuals had polygenic scores that were higher than the 90^th^ percentile of the population for LDL-C and TG, respectively. In 13 families three or more affected individuals had high polygenic score and in 22 families none of the affected individuals had polygenic score above the 90^th^ percentile of the population.

Our results allow us to draw several conclusions about the genetic background of FCH. First, variants across the whole frequency spectrum were enriched and contribute to the high LDL-C and/or TG levels in the affected FCH individuals. This emphasizes the polygenic nature of FCH and is in line with previous results with familial hypercholesterolemia demonstrating that this less prevalent syndrome has a polygenic component [9]. Genes in which we observed enriched variants include *APOE*, *LIPC* and *APOA5*, whose role in dyslipidemias is already established, but also include several genes whose function in lipid metabolism is not yet clear (*e*.*g*. *UBR1*, *MTHFD2L* and the *PIGV-NR0B2* region).

Second, the majority of the enriched variants were originally identified in random population samples. This finding confirms that variants identified in population screens play a considerable role also in the complex familial disease FCH, and highlights the potential of using variants identified in population-based GWA studies to characterize familial dyslipidemia cases genetically.

Third, the observed enrichment was stronger for TG loci than for LDL-C loci. Many of the enriched TG SNPs were located in genes known to contribute to hypertriglyceridemia, such as the *APOA1-C3-A4-A5* cluster, showing the central role of genetically driven TG in FCH [7, 17].

Fourth, over a third of the affected FCH individuals had a high load (polygenic lipid scores over the 90^th^ percentile of the population) of either known LDL-C or TG associated variants. This proportion with high polygenic score was comparable to the population samples with similar levels of high LDL-C or TG in the population. This observation suggests that the role of the known variants is similar in familial hyperlipidemias and randomly ascertained high LDL-C and/or TG individuals. However, the accumulation of LDL-C or TG-elevating alleles in families was highlighted by the prediction analysis results. High load of known LDL-C or TG associated variants predicted affectedness in FCH families almost two times better than comparable hyperlipidemia in the population.

There were large differences among families in the proportion of affected individuals with high polygenic scores. While in nine families more than two thirds of the affected individuals had high polygenic score, in over a third of the families, none of the cases had high score and the underlying genetic architecture remains unexplained in the majority of families. As genotyping and imputation allow for the evaluation of only a portion of catalogued high-impact variants, future sequencing studies might pinpoint rare causal variants in these families.

Our study supports the hypothesis that FCH is a genetically heterogeneous disease, reflected by its heterogeneous lipid phenotype [18–20]. large number of LDL-C associated variants are mainly responsible for the elevation of LDL-C in the FCH lipid profile. In contrast, a handful of low- to moderate-impact TG variants drive the elevation of triglycerides. Our study provides support for the polygenic rather than monogenic nature of FCH, and highlights the central role of genetically driven TG in FCH.

## Materials and Methods

### Ethics statement

Written informed consent was obtained from all study participants. All samples were collected in accordance with the Helsinki declaration and study protocols were approved by the ethics committees of the participating centers (The Hospital District of Helsinki and Uusimaa Coordinating Ethics Committee, approval number 184/13/03/00/12).

### Subjects and measurements

As part of the European Multicenter Study on Familial Dyslipidemias in Patients with Premature Coronary Heart Disease (EUFAM), the Finnish FCH families were identified from patients admitted to university hospitals with a diagnosis of premature CHD who demonstrated levels of TC, TG, or both that were ≥ 90^th^ Finnish age- and sex-specific population percentile (S4 Table) [21, 22]. Families that contained at least one other first-degree relative affected with hyperlipidemia (according to the same lipid-level criteria as used for the probands) were included in the study. Additionally, in the included families, at least one of the affected individuals had high TG. All family members with hyperlipidemia were then considered affected by FCH. Probands with a diagnosis of FH (screened with a functional LDL receptor test), and any subjects with diabetes or other chronic diseases were considered unaffected and did not contribute to establishing the family’s FCH status (S1 Text).

For analyses of continuous lipid traits, individuals using lipid-lowering or estrogen medication at the time of sampling were excluded. Samples from the Finnish National FINRISK study were used as a Finnish population-specific comparison group, and individuals with known diabetes or cancer were excluded from the analyses (S1 Text). For the FCH families, venous blood samples were obtained after an overnight fast. FINRISK participants were advised to fast for four hours before the examination and avoid heavy meals earlier during the day. For both the EUFAM and FINRISK samples, circulating biochemical markers were measured from the venous blood samples using standard methods, as described in the S1 Text.

### Genotyping and imputation

All FCH individuals with DNA available (715 out of 1161 from all 53 FCH families) and the FINRISK samples (*n* = 20,626) were genotyped with the HumanCoreExome BeadChip (Illumina Inc., San Diego, CA, USA) using standard methods (S1 Text). Additionally, over nine million variants were imputed using a combined reference panel of 1000 Genomes Phase I integrated haplotypes and 1943 Finnish genomes (S1 Text).

### Polygenic lipid score calculation

To construct a polygenic lipid score, we catalogued and used 212 SNPs, representing either the lead SNPs for genome-wide significant associations to LDL-C or TG from GWA studies of these traits (171 SNPs), or SNPs catalogued in the Online Mendelian Inheritance in Man (OMIM) database as located in genes implicated in primary and secondary monogenic dyslipidemic syndromes (44 SNPs, four of which overlap with the GWA lead-SNPs, S2 Table) [23–26]. The SNPs were assigned to LDL-C and TG scores based on their previously reported associations (S1 Text). The scores were calculated as the sum of the risk alleles weighted by their effect estimates drawn from a multiple linear model estimating all SNP effects on the trait (LDL-C or TG) at the same time in the FINRISK samples (S1 Text). The FINRISK samples used to estimate the weights were independent from the FCH samples.

### Statistical analysis

We estimated allele frequencies for all 212 SNPs in the 234 affected FCH individuals and in 18,715 FINRISK samples after excluding individuals with known diabetes or cancer similarly to the EUFAM exclusion criteria. The minor allele in FINRISK was designated as the effect allele. We then calculated enrichment ratios by dividing the effect allele frequencies in affected FCH individuals with the effect allele frequencies in the FINRISK samples. Under the null hypothesis of no enrichment, we estimated the null distribution for enrichment testing by calculating the enrichment statistic (enrichment ratio) for all variants with MAF > 0.001% (12,234,754 SNPs) across the genome excluding regions within 50 000 base pairs from the lipid-associated SNPs. We estimated the null distribution in MAF bins across the genome while keeping the family structure fixed (FINRISK minor allele frequency intervals [0.1%,0.5%); [0.5%,1%); [1%,1.5%); [1.5%,2%); [2%,2.5%), [2.5%,5%); [5%,10%); [10%,15%); [15%,20%); [20%,25%); [25%,30%); [30%,35%); [35%,40%); [40%,45%); and [45%,50%)). The observed lipid SNP allele frequency enrichment ratios were compared to the null distribution to provide one-sided *p*-values for each SNP. We used sign test for all SNPs together to estimate whether the direction of enrichment (more or less common in the affected FCH individuals than in the population) was associated with the SNPs’ effects on the lipid in question (elevating or lowering).

We applied linear mixed models to test for differences in metabolic and clinical characteristics between FCH affected and unaffected individuals (S1 Text). An empirical genetic correlation matrix between individuals was included as the covariance structure of a random effect. Linear mixed models were applied with MMM (version 1.01) [27], and the other statistical analyses were performed using R (version 3.2.1) [28].

## Supporting Information

S1 Text Supplementary Materials and Methods. The recruitment, assessment, and genotyping of study subjects, the calculation of polygenic lipid scores, and the statistical analyses performed are described in more detail.
(DOCX)

S1 Fig Number of affected and unaffected individuals in FCH families. The number of genotyped affected (black) and unaffected individuals (white) is presented for each FCH family. The families are sorted by the number of affected individuals in each family, but the family numbers on the x-axis correspond to those presented in Fig 3.
(PDF)

S2 Fig Lipid level median values and ranges in FCH families in genotyped individuals. Median values of (a) LDL-C and (b) TG for genotyped individuals in all families (*n* = 53). Vertical lines represent the range of values within the families. The families are sorted by the medians of LDL-C and TG levels, respectively, but the family numbers on the x-axis correspond to those presented in Fig 3. In (b) the y-axis is cut at 7 mmol/l.
(PDF)

S3 Fig Enrichment of LDL-C or TG associated SNPs in FCH probands and all FCH family members by their frequency and effect on LDL-C and TG levels. Enrichment ratio is the ratio of effect allele frequency in FCH probands (*n* = 48, (a), (b)), or in all FCH family members (n = 715, (c), (d)), to allele frequencies in the Finnish FINRISK population cohort (*n* = 20,626 in c) and d); *n* = 18,715 in a) and b) after excluding individuals with diabetes and cancer). Under the null hypothesis of no enrichment, a 95% credible interval (shaded area) was estimated by calculating the enrichment statistic (enrichment ratio) for all variants with MAF > 0.001% across the genome excluding the loci of the 212 SNPs. Variants are designated as either lipid level elevating (red) or lowering (blue) for LDL-C ((a), (c)) and TG ((b), (d)) based on *β* estimates from linear regression in the FINRISK samples. Point size and color intensity reflect the magnitude of the effect. Only SNPs with at least one heterozygous carrier are shown (*n* = 191 for probands and *n* = 196 for all family members). FINRISK, The National FINRISK Study. *The enrichment ratio for *LPL* rs1801177 fell within the 95% credible interval.
(PDF)

S4 Fig Triglyceride levels in carriers and wild type individuals of *APOA5* rs3135506 and *APOE* rs7412. Homozygosity for (a) *APOA5* rs3135506 predisposes to hypertriglyceridemia and homozygosity for (b) *APOE* rs7412 predisposes to type III hyperlipoproteinemia, which typically presents in elevated levels of VLDL and TG species. Only individuals without diabetes and other relevant confounders were included in this comparison. In (b) the y-axis is log-scaled.
(PDF)

S5 Fig Mean genetic correlation between affected family members in FCH families. For each family with at least two genotyped affected members (*n* = 51), we calculated mean empiric genetic correlation of all combinations of pairs of affected subjects. Families are ranked on the x-axis as in Fig 3, with higher ranking representing a higher number of affected subjects with high polygenic lipid scores or high-impact Mendelian variants. A significance estimate for the relationship was derived from simple linear regression.
(PDF)

S1 Table Clinical and metabolic characteristics of all individuals. *P-*values were calculated using Wald test by a linear mixed model correcting for sample relatedness. FCH, familial combined hyperlipidemia.
(PDF)

S2 Table LDL-C or TG associated variants included in the study. NFE, non-Finnish Europeans; FINRISK, The National FINRISK Study; FCH, familial combined hyperlipidemia. *Effect allele. ^†^Global minor allele. ^‡^MAF in non-Finnish Europeans calculated from the public ExAC data set (version 0.3) for coding SNPs (those present in the ExAC data set), and from the public 1000 Genomes Phase 3 data set for non-coding SNPs (S1 Text). ^§^MAF in the FINRISK cohort after excluding those with known diabetes or cancer. ^∥^Common: MAF > 5%, low-frequency: 0.5% < MAF ≤ 5%, rare: MAF ≤ 0.5%. ^¶^SNPs were selected based on OMIM entries for genes implicated in monogenic dyslipidemia, and recent genome-wide association studies (S1 Text). ^♦^Effect allele weight not estimated due to only one copy of minor allele present in FINRISK.
(PDF)

S3 Table Enrichment ratios and observed *p*-values for all score SNPs with at least one affected carrier. Under the null hypothesis of no enrichment, we established the null distribution for enrichment testing by calculating the enrichment statistic (enrichment ratio) for all variants with effect allele (minor allele in the FINRISK cohort) frequency > 0.001% across the genome excluding the loci of the 212 SNPs. Enrichment ratios were calculated for each of the 212 SNPs if at least one carrier was identified (194 SNPs for affected individuals, 191 SNPs for probands), and one-tailed *p*-values were estimated from the null distribution. Enrichment ratios are presented for the affected individuals. The carrier counts were calculated from all genotyped FCH family members who passed exclusion criteria and did not have diabetes or other relevant confounders (*n* = 661), and did not account for non-independence within family. The + and—symbols denote elevating and lowering effects on the lipid in question, respectively. FCH, familial combined hyperlipidemia; FINRISK, The National FINRISK Study. J.A.K, J. A. Kuivenhoven; C.J.W., C. J. Willer; T.M.T., T. M. Teslovich; I.S., I. Surakka (S1 Text). ^†^Effect allele frequencies in non-Finnish Europeans were calculated from the public ExAC data set (version 0.3) for coding SNPs (those present in the ExAC data set), and from the public 1000 Genomes Phase 3 data set for non-coding SNPs (S1 Text). ^‡^Effect allele frequencies in the FINRISK cohort after excluding those with known diabetes or cancer. ^§^Enrichment ratio is the ratio of the effect allele frequencies in affected individuals (*n* = 234) to the Finnish population (*n* = 18,715).
(PDF)

S4 Table The 90^th^ age- and sex-specific Finnish population percentiles for total cholesterol and triglycerides. In the EUFAM study, dyslipidemia was established based on levels of total cholesterol, triglycerides, or both that were ≥ 90th Finnish age- and sex-specific population percentile. The population percentiles were derived from FINMONICA, a large population survey performed in 1992. The percentile estimates and a description of the polynomial regression analyses used to establish them have been reported in detail previously [21].
(PDF)
