# Supplementary material for: The Contribution of GWAS Loci in Familial Dyslipidemias
Source: PLoS Genet. 2016 May 26;12(5):e1006078. doi: 10.1371/journal.pgen.1006078 (PMC4882070; doi:10.1371/journal.pgen.1006078)
Supplement: S1 Text — The recruitment, assessment, and genotyping of study subjects, the calculation of polygenic lipid scores, and the statistical analyses performed are described in more detail. (DOCX) [file pgen.1006078.s001.docx]

# S1 Appendix. Supplementary Materials and Methods.

## Patients

The European Multicenter Study on Familial Dyslipidemias in Patients with Premature Coronary Heart Disease (EUFAM) aims to resolve metabolic and genetic abnormalities of familial dyslipidemias predisposing to coronary heart disease (CHD) [[1](#_ENREF_1)]. As part of the EUFAM study, the Finnish familial combined hyperlipidemia (FCH) families have been identified via proband individuals admitted to Finnish university hospitals with a diagnosis of abnormally premature CHD (30 years to 60 years of age and myocardial infarction [MI] or > 50 % stenosis in at least one coronary artery) and dyslipidemia. If also at least one first-degree relative filling criteria of dyslipidemia could be identified, invitation was extended to all other relatives and spouses in the pedigree.

The probands were assigned a dyslipidemia diagnosis based on their measured blood lipid profile in relation to age and sex specific Finnish population percentiles observed in the National FINRISK Study (FINRISK) [[2](#_ENREF_2)]. FCH was diagnosed if total cholesterol (TC), triglycerides (TG), or both were ≥ 90^th^ age and sex-specific population percentile (S4 Table). Common exclusion criteria for probands were diabetes (unless verifiably preceded by CHD), hepatic or renal disease, hypo- or hyperthyroidism, pregnancy, and malignancies. Also, families of probands without any glucose data were excluded from the study. To exclude individuals with classic familial hypercholesterolemia (FH), probands were screened with an in-house functional low-density lipoprotein (LDL) receptor test similar to a test developed by Cuthbert and colleagues [[3](#_ENREF_3)]. Family members were considered affected by FCH if they passed the exclusion criteria and fulfilled the same blood lipid profile criteria as the proband, otherwise they were considered unaffected. Families with at least one affected first-degree relative, including at least one family member with high TG (to exclude isolated hypercholesterolemia), were included in the study.

The Finnish population control samples from the National FINRISK study were collected as described [[2](#_ENREF_2)].

## Measurements

For the FCH families, venous blood samples were obtained after an overnight fast. Serum and EDTA plasma were separated by centrifugation and stored at -80 °C until analysis with no LCAT inhibitors added. Height (to the nearest 0.5 cm) and weight (to the nearest 0.1 kg with light clothing and without shoes) were recorded. Body mass index (BMI) was calculated as weight/height^2^ (kg/m^2^).

Serum TC and TG were determined with an automated Cobas Mira analyzer (Hoffman-La Roche, Basel, Switzerland) by fully enzymatic methods (Hoffman-La Roche kits #0722138 and #0715166, respectively). Serum high-density lipoprotein cholesterol (HDL-C) was quantified by phosphotungstic acid/magnesium chloride precipitation procedures (Hoffman-La Roche kit #0720674). Serum LDL-C was calculated using the Friedewald formula [[4](#_ENREF_4)]. Very low-density lipoprotein, LDL, and intermediate-density lipoprotein compositions were analyzed as described [[5](#_ENREF_5)].

Apolipoprotein E (apoE) isoform was phenotyped by isoelectric focusing of serum [[6](#_ENREF_6)]. The *APOE* ε2/ε3/ε4 haplotype, which determines the apoE isoform and observed phenotype, is defined by two SNPs (rs429358 and rs7412). In our material, we genotyped rs7412 and imputed rs429358 and compared the participants’ genetic haplotype with their observed phenotype (Table 3) [[7](#_ENREF_7)]. The *APOE* haplotype of a subject was determined only if the posterior probability of rs429358 was > 0.9 for any allele.

Measurements for the FINRISK samples were obtained as described [[2](#_ENREF_2)].

# Genotyping, quality control, and imputation

DNA samples from 715 individuals were genotyped in University of California, Los Angeles (UCLA) using the HumanCoreExome BeadChip (Illumina Inc., San Diego, CA, USA), a high-resolution genotyping array with all the approximately 260,000 tag SNPs from the HumanCore BeadChip plus approximately 250,000 exonic markers from the HumanExome BeadChip. Genotype calls were generated together with other available HumanCoreExome data sets using zCall at FIMM. After quality control (sample call rate > 98 %, SNP call rate > 95 % [> 99 % if minor allele frequency {MAF} < 1 %], removal of SNPs with Hardy-Weinberg equilibrium *p* < 10^-6^ [in Finnish population data], incoherent allele frequencies or minor allele count < 2, removal of outliers in heterozygosity [±3 SD from mean], removal of samples with unclear sex, removal of duplicates, removal of three outlier subjects based on MDS plot, resetting of genotypes with Mendel errors), the samples were phased using SHAPEIT (version 2) [[8](#_ENREF_8)] and imputed with IMPUTE (version 2.3.1) [[8-10](#_ENREF_8)] and did not take into account family relationships. The reference panel used was a combination of 1000 Genomes Phase I integrated haplotypes produced using SHAPEIT (version 2) release June 2014 and an in-house reference panel with 1941 whole genome sequenced Finnish individuals from Finnish FINRISK and Health 2000 population cohorts [[11](#_ENREF_11)]. Finally, all 715 samples from FCH families were successfully imputed.

FINRISK samples (*n* = 20,626) were genotyped using the same chip and imputed using the same reference panel as the FCH family members described above using standard methods [[2](#_ENREF_2)].

## Polygenic lipid score calculation

We used all genome-wide significantly (*p* < 5*x*10^-8^) LDL-C- or TG-associated lead-SNPs from the three most recent and extensive lipid-level genome-wide association screens as well as SNPs catalogued in the Online Mendelian Inheritance in Man (OMIM) database for genes implicated in primary and secondary monogenic dyslipidemias as the basis of our polygenic lipid score calculation (S2 Table) [[12-15](#_ENREF_12)]. Limiting the SNPs to those observable and varying in our imputed genotypes resulted in 212 SNPs, out of which 114 were directly genotyped and 98 imputed. SNPs that were genome-wide significantly associated with LDL-C were included in the LDL-C score and SNPs associated with TG in the TG score. The OMIM SNPs were included in both scores, unless they had a genome-wide significant *p*-value to either lipid in any of the GWA studies, in which case they were only included in that lipid’s score.

For each individual and lipid, the polygenic lipid scores were calculated using the weighted sum of the risk alleles. The weights used were estimated using multiple linear regression including age, sex, genotyping platform, and project year together with the score-specific SNPs (141 for LDL-C, 116 for TG) resulting in uncorrelated effect estimates for the SNPs. Lipid values were natural logarithm transformed for the model and genotypes were used as allelic dosages, i.e. 0, 1 or 2 for the genotyped SNPs, or expected number of allele based on the imputation posterior probability for the imputed SNPs. The effect estimation was performed using the FINRISK cohort of some 19,000 (18,834 for LDL-C and 19,114 for TG) Finnish individuals not on lipid medication imputed using the same reference panel as our samples [[2](#_ENREF_2)]. The imputed SNPs were also weighted by their posterior probabilities. We used R (version 3.2.1) to calculate the scores [[16](#_ENREF_16)].

## Statistical analysis

We applied a linear mixed model to test for differences in metabolic and clinical characteristics between FCH affected and unaffected individuals (Table 1 and S1 Table). We included an indicator variable of being affected as a fixed effect in the model and evaluated its *p*-value using a Wald test. Relatedness of the samples was accounted for by including a random effect in the model. The covariance matrix of the random effect was an empirical genome-wide genetic correlation matrix when comparing genotyped individuals, and a theoretical kinship matrix based on the pedigree data when comparing all individuals. The outcome variable was being male in male/female comparison, smoking status in number of individuals smoking, and the values of the measurements in other continued measures. The linear mixed models were applied with MMM (version 1.01) [[17](#_ENREF_17)].

## Allele Frequencies in the population

Allele frequencies in non-Finnish Europeans were estimated from the public ExAC data set (version 0.3) for coding SNPs (those present in the ExAC data set), and from the public 1000 Genomes Phase 3 data set for non-coding SNPs [[18-21](#_ENREF_18)]

## References

1. Porkka KV, Nuotio I, Pajukanta P, Ehnholm C, Suurinkeroinen L, Syvanne M, et al. Phenotype expression in familial combined hyperlipidemia. Atherosclerosis. 1997;133(2):245-53.

2. Borodulin K, Vartiainen E, Peltonen M, Jousilahti P, Juolevi A, Laatikainen T, et al. Forty-year trends in cardiovascular risk factors in Finland. Eur J Public Health. 2015;25(3):539-46. doi: 10.1093/eurpub/cku174.

3. Cuthbert JA, East CA, Bilheimer DW, Lipsky PE. Detection of familial hypercholesterolemia by assaying functional low-density-lipoprotein receptors on lymphocytes. N Engl J Med. 1986;314(14):879-83. doi: 10.1056/NEJM198604033141404.

4. Friedewald WT, Levy RI, Fredrickson DS. Estimation of the concentration of low-density lipoprotein cholesterol in plasma, without use of the preparative ultracentrifuge. Clin Chem. 1972;18(6):499-502.

5. Taskinen MR, Kuusi T, Helve E, Nikkila EA, Yki-Jarvinen H. Insulin therapy induces antiatherogenic changes of serum lipoproteins in noninsulin-dependent diabetes. Arteriosclerosis. 1988;8(2):168-77.

6. Lehtimaki T, Moilanen T, Viikari J, Akerblom HK, Ehnholm C, Ronnemaa T, et al. Apolipoprotein E phenotypes in Finnish youths: a cross-sectional and 6-year follow-up study. J Lipid Res. 1990;31(3):487-95.

7. Tejedor MT, Garcia-Sobreviela MP, Ledesma M, Arbones-Mainar JM. The Apolipoprotein E Polymorphism rs7412 Associates with Body Fatness Independently of Plasma Lipids in Middle Aged Men. PLoS One. 2014;9(9):e108605. doi: 10.1371/journal.pone.0108605.

8. Delaneau O, Marchini J, Zagury JF. A linear complexity phasing method for thousands of genomes. Nature methods. 2012;9(2):179-81. doi: 10.1038/nmeth.1785.

9. Howie B, Marchini J, Stephens M. Genotype imputation with thousands of genomes. G3. 2011;1(6):457-70. doi: 10.1534/g3.111.001198.

10. Howie BN, Donnelly P, Marchini J. A flexible and accurate genotype imputation method for the next generation of genome-wide association studies. PLoS Genet. 2009;5(6):e1000529. doi: 10.1371/journal.pgen.1000529.

11. Genomes Project C, Abecasis GR, Auton A, Brooks LD, DePristo MA, Durbin RM, et al. An integrated map of genetic variation from 1,092 human genomes. Nature. 2012;491(7422):56-65. doi: 10.1038/nature11632.

12. Kuivenhoven JA, Hegele RA. Mining the genome for lipid genes. Biochim Biophys Acta. 2014;1842(10):1993-2009. doi: 10.1016/j.bbadis.2014.04.028.

13. Teslovich TM, Musunuru K, Smith AV, Edmondson AC, Stylianou IM, Koseki M, et al. Biological, clinical and population relevance of 95 loci for blood lipids. Nature. 2010;466(7307):707-13. Epub 2010/08/06. doi: 10.1038/nature09270.

14. Surakka I, Horikoshi M, Magi R, Sarin AP, Mahajan A, Lagou V, et al. The impact of low-frequency and rare variants on lipid levels. Nat Genet. 2015;47(6):589-97. doi: 10.1038/ng.3300.

15. Global Lipids Genetics Consortium, Willer CJ, Schmidt EM, Sengupta S, Peloso GM, Gustafsson S, et al. Discovery and refinement of loci associated with lipid levels. Nat Genet. 2013;45:1274-83. doi: 10.1038/ng.2797.

16. R Core Team. R: A Language and Environment for Statistical Computing. R Foundation for Statistical Computing; 2014.

17. Pirinen M, Donnelly P, Spencer CCA. Efficient computation with a linear mixed model on large-scale data sets with applications to genetic studies. Ann Appl Stat. 2013;7(1):369-90. doi: 10.1214/12-AOAS586.

18. Sudmant PH, Rausch T, Gardner EJ, Handsaker RE, Abyzov A, Huddleston J, et al. An integrated map of structural variation in 2,504 human genomes. Nature. 2015;526(7571):75-81. doi: 10.1038/nature15394.

19. 1000 Genomes Phase 3 Release [cited 2015 13/10]. Available from: <http://ftp.1000genomes.ebi.ac.uk/vol1/ftp/release/20130502/>.

20. Exome Aggregation Consortium (ExAC), Cambridge, MA (URL: <http://exac.broadinstitute.org>) [2, 2015].

21. Lek M, Karczewski K, Minikel E, Samocha K, Banks E, Fennell T, et al. Analysis of protein-coding genetic variation in 60,706 humans. Preprint. bioRxiv. 2015. doi: 10.1101/030338.
