# Supplementary figures and images for: The Contribution of GWAS Loci in Familial Dyslipidemias
Source: PLoS Genet. 2016 May 26;12(5):e1006078. doi: 10.1371/journal.pgen.1006078 (PMC4882070; doi:10.1371/journal.pgen.1006078)

Number of individuals

30

20

10

0

2 6 1 4 14 15 16 20 25 28 31 38 3 8 12 13 26 30 34 35 44 5 7 22 24 33 36 37 48 9 19 21 23 29 32 39 43 46 47 50 11 27 40 10 41 42 49 53 17 18 45 52 51

■ Affected individuals ▨ Unaffected individuals

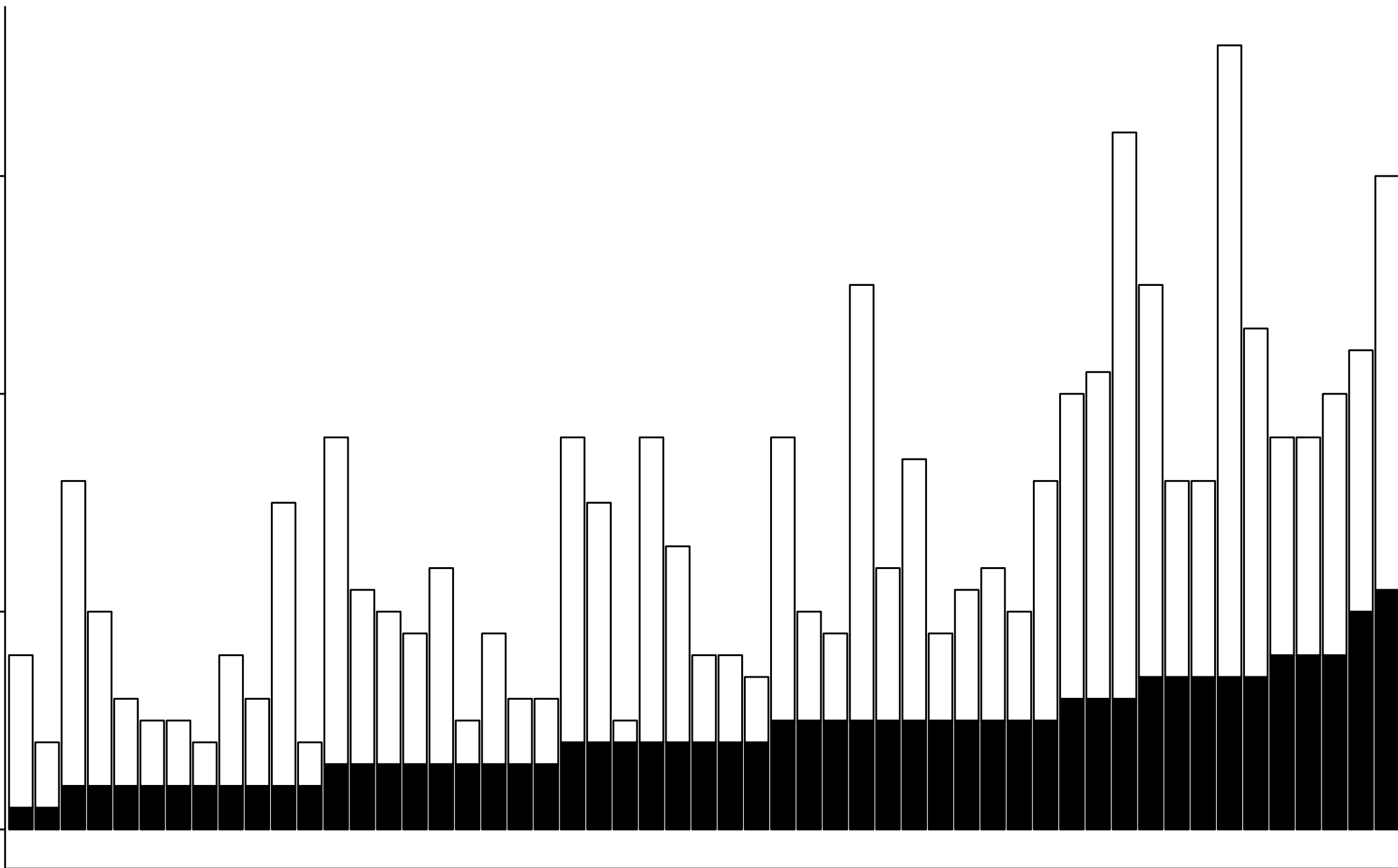

Supplement: S1 Fig — The number of genotyped affected (black) and unaffected individuals (white) is presented for each FCH family. The families are sorted by the number of affected individuals in each family, but the family numbers on the x-axis correspond to those presented in Fig 3. (PDF) [file pgen.1006078.s002.pdf]

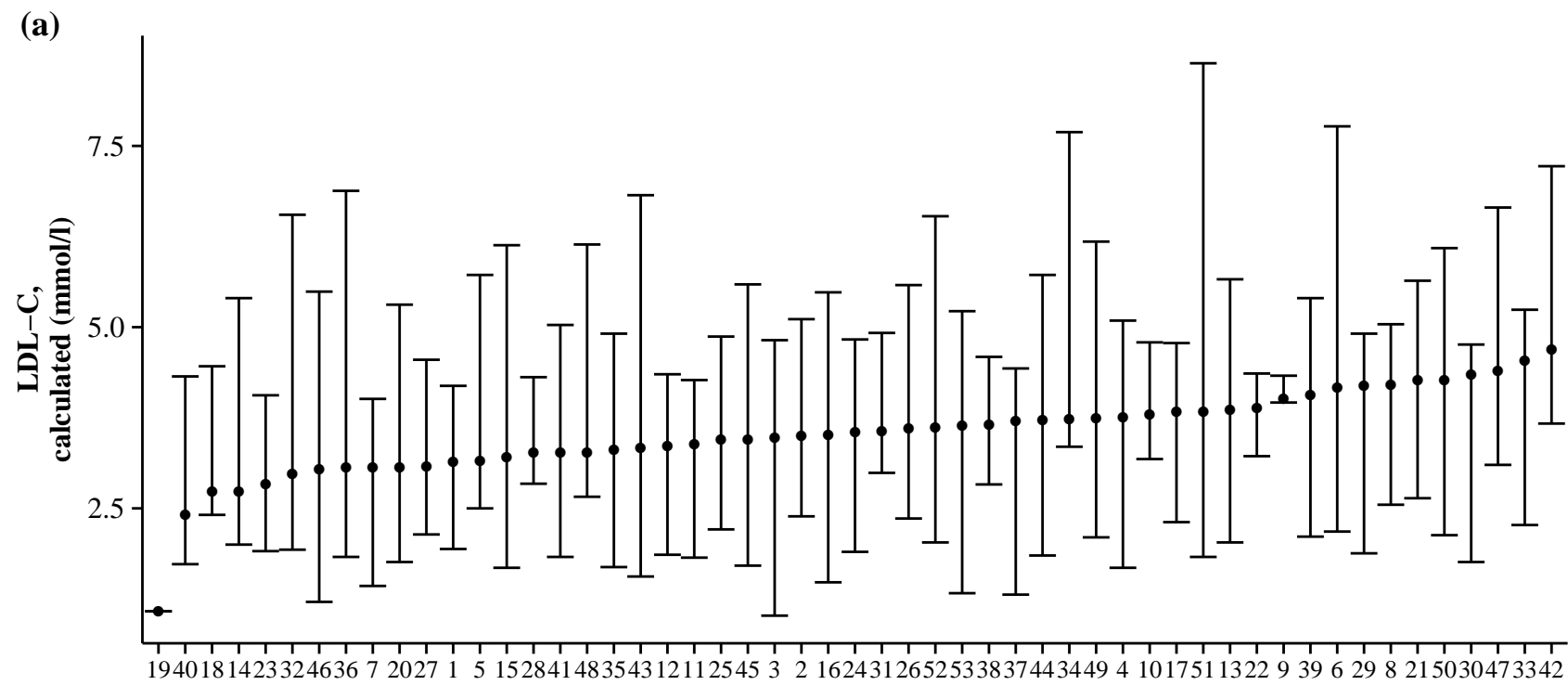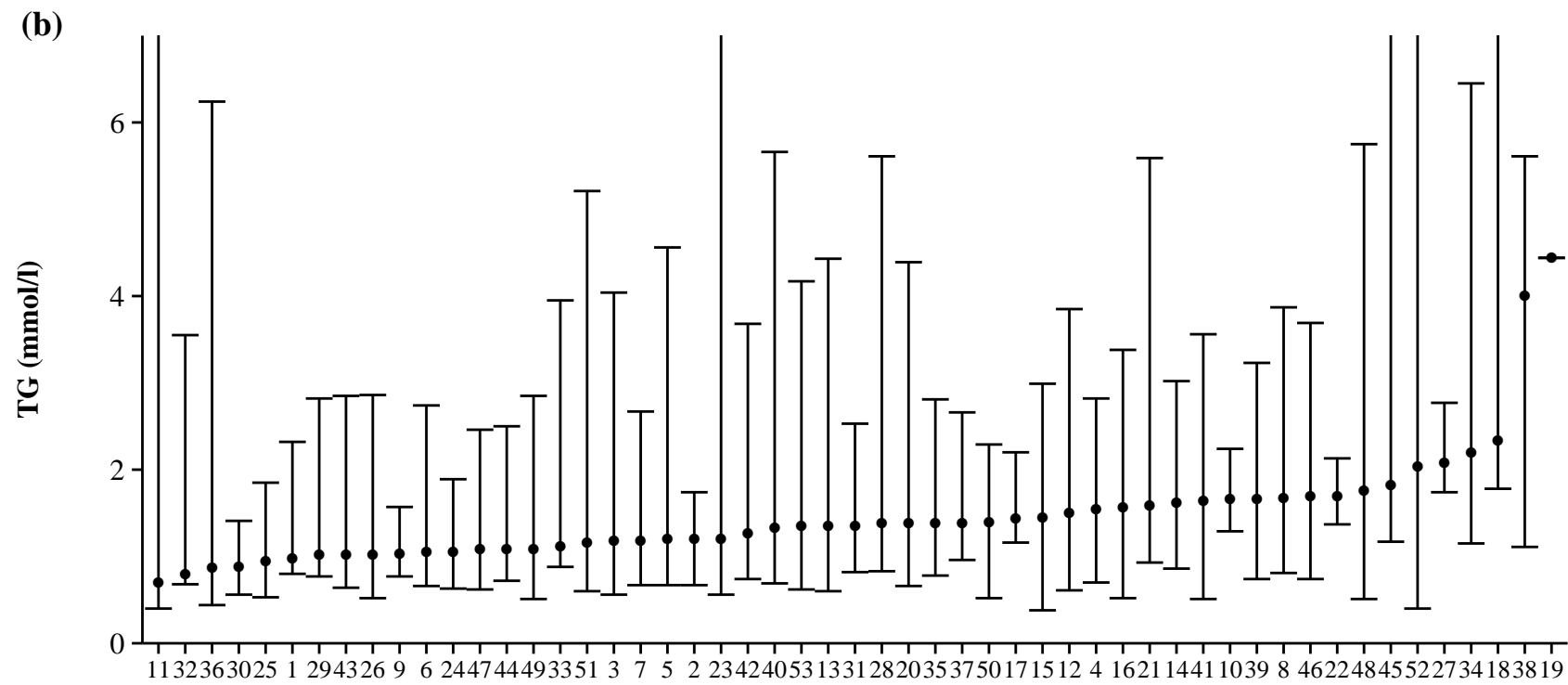

Supplement: S2 Fig — Median values of (a) LDL-C and (b) TG for genotyped individuals in all families (n = 53). Vertical lines represent the range of values within the families. The families are sorted by the medians of LDL-C and TG levels, respectively, but the family numbers on the x-axis correspond to those presented in Fig 3. In (b) the y-axis is cut at 7 mmol/l. (PDF) [file pgen.1006078.s003.pdf]

(a)

**APOA5 rs3135506  
in FCH pedigrees**

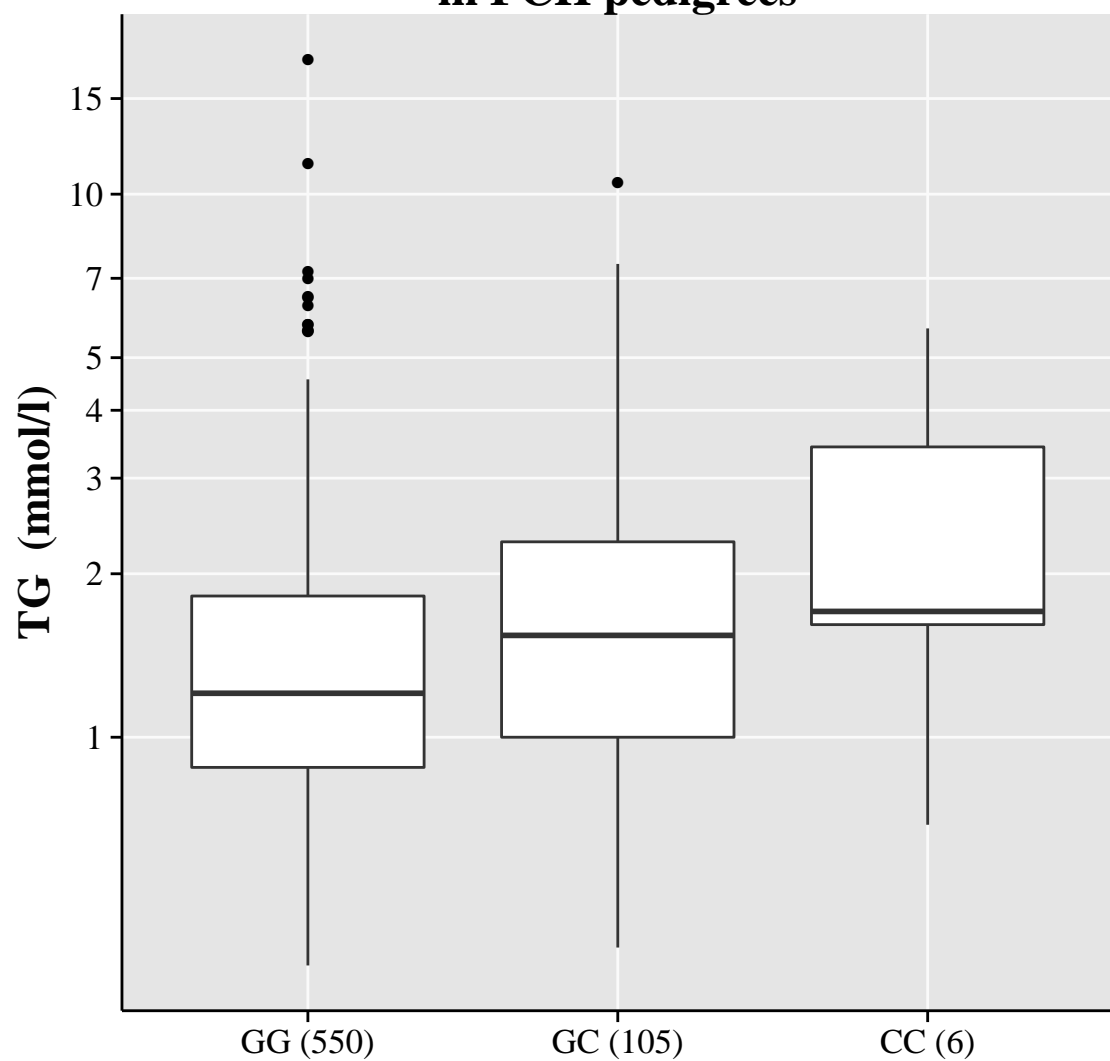

(b)

**APOE rs7412  
in FCH pedigrees**

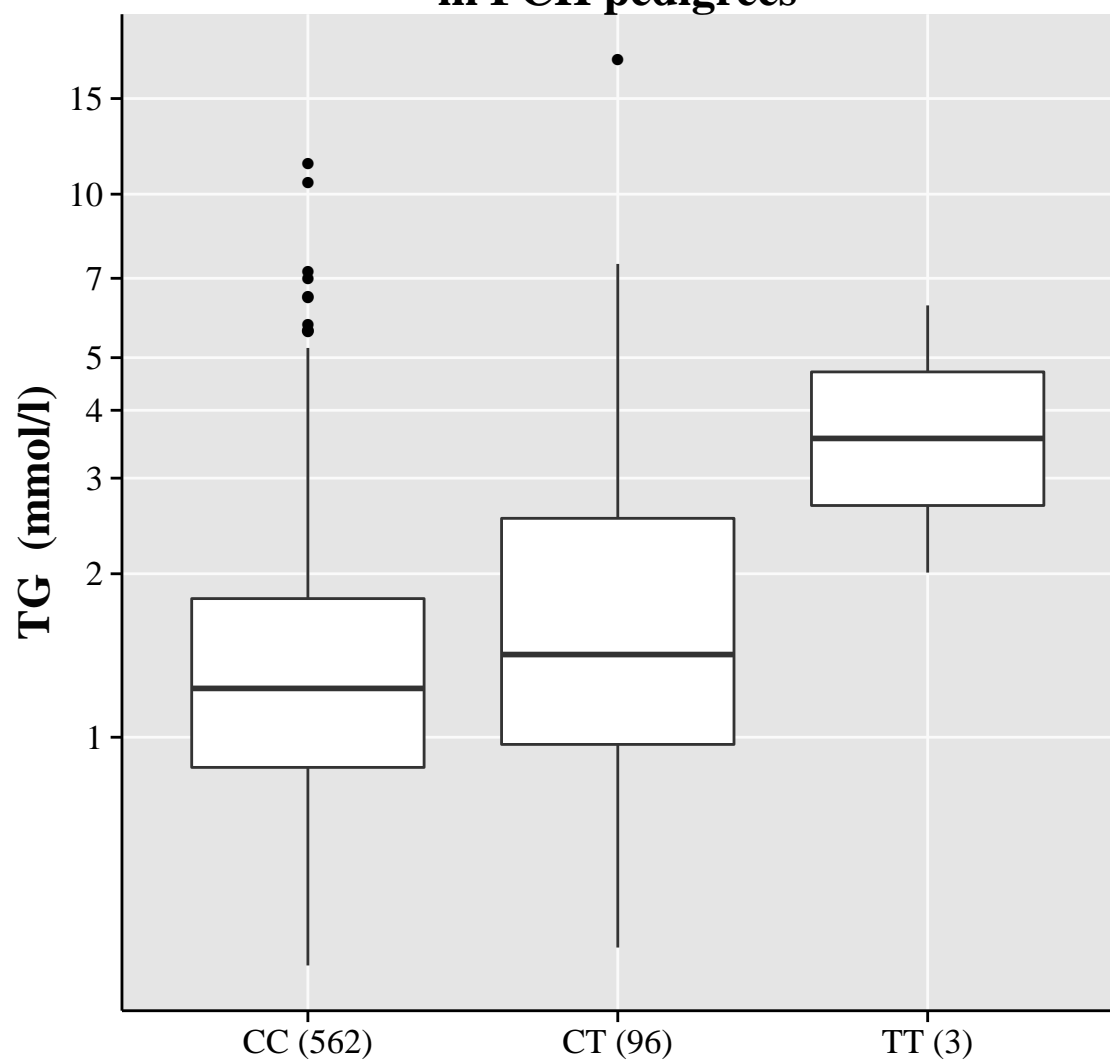

Supplement: S4 Fig — Homozygosity for (a) APOA5 rs3135506 predisposes to hypertriglyceridemia and homozygosity for (b) APOE rs7412 predisposes to type III hyperlipoproteinemia, which typically presents in elevated levels of VLDL and TG species. Only individuals without diabetes and other relevant confounders were included in this comparison. In (b) the y-axis is log-scaled. (PDF) [file pgen.1006078.s005.pdf]

Mean genetic correlation between affected FCH family members

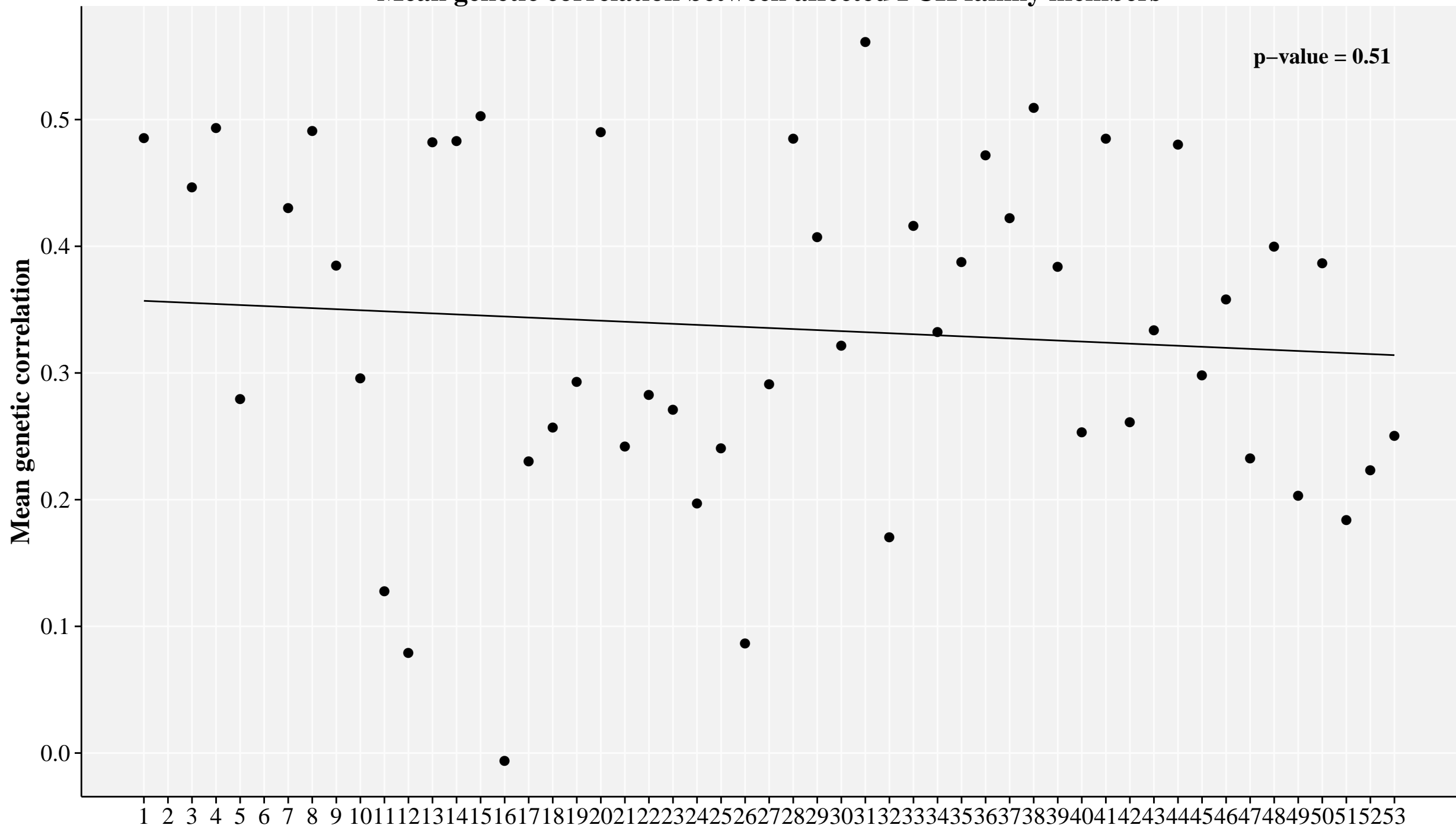

Supplement: S5 Fig — For each family with at least two genotyped affected members (n = 51), we calculated mean empiric genetic correlation of all combinations of pairs of affected subjects. Families are ranked on the x-axis as in Fig 3, with higher ranking representing a higher number of affected subjects with high polygenic lipid scores or high-impact Mendelian variants. A significance estimate for the relationship was derived from simple linear regression. (PDF) [file pgen.1006078.s006.pdf]
