# Supplementary material for: The Contribution of GWAS Loci in Familial Dyslipidemias
Source: PLoS Genet. 2016 May 26;12(5):e1006078. doi: 10.1371/journal.pgen.1006078 (PMC4882070; doi:10.1371/journal.pgen.1006078)
Supplement: S3 Fig — Enrichment ratio is the ratio of effect allele frequency in FCH probands (n = 48, (a), (b)), or in all FCH family members (n = 715, (c), (d)), to allele frequencies in the Finnish FINRISK population cohort (n = 20,626 in c) and d); n = 18,715 in a) and b) after excluding individuals with diabetes and cancer). Under the null hypothesis of no enrichment, a 95% credible interval (shaded area) was estimated by calculating the enrichment statistic (enrichment ratio) for all variants with MAF > 0.001% across the genome excluding the loci of the 212 SNPs. Variants are designated as either lipid level elevating (red) or lowering (blue) for LDL-C ((a), (c)) and TG ((b), (d)) based on β estimates from linear regression in the FINRISK samples. Point size and color intensity reflect the magnitude of the effect. Only SNPs with at least one heterozygous carrier are shown (n = 191 for probands and n = 196 for all family members). FINRISK, The National FINRISK Study. *The enrichment ratio for LPL rs1801177 fell within the 95% credible interval. (PDF) [file pgen.1006078.s004.pdf]

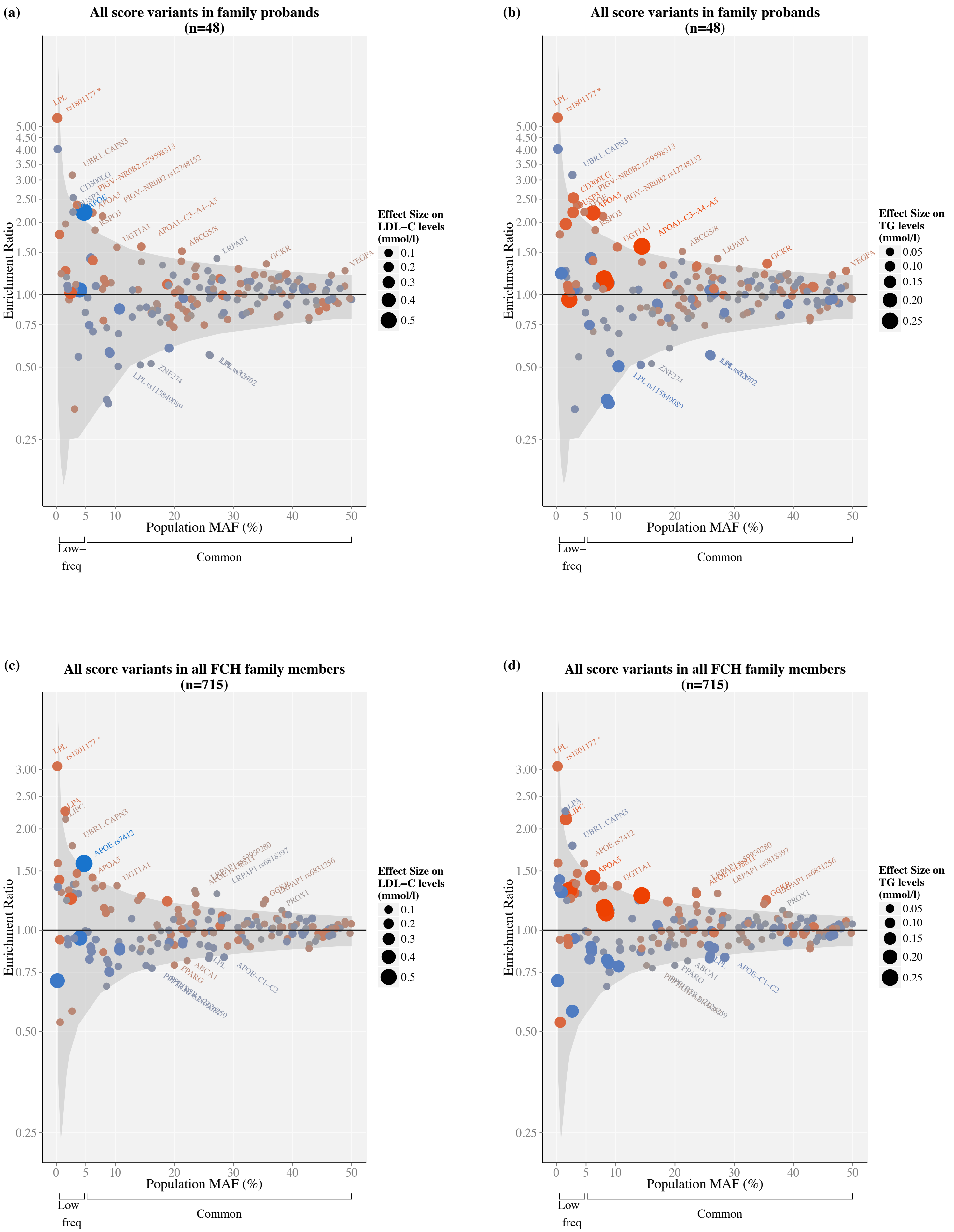
(a)
All score variants in family probands
(n=48)
(b)
All score variants in family probands
(n=48)
(c)
All score variants in all FCH family members
(n=715)
(d)
All score variants in all FCH family members
(n=715)
Enrichment Ratio
Population MAF (%)
Low–
freq
Common
Effect Size on
LDL–C levels
(mmol/l)
0.1
0.2
0.3
0.4
0.5
Effect Size on
TG levels
(mmol/l)
0.05
0.10
0.15
0.20
0.25
LPL
rs1801177 *
UBR1, CAPN3
CD300LG
PIGV–NR0B2 rs79598313
PIGV–NR0B2 rs12748152
APOA5
APOE
RSPO3
UGT1A1
APOA1–C3–A4–A5
ABCG5/8
LRPAP1
GCKR
VEGFA
ZNF274
LPL rs115849089
LPA
LIPC
APOE rs7412
LRPAP1 rs6818397
LRPAP1 rs6831256
PROX1
ABCA1
PPARG
APOE–C1–C2
