# Supplementary material for: The Contribution of GWAS Loci in Familial Dyslipidemias
Source: PLoS Genet. 2016 May 26;12(5):e1006078. doi: 10.1371/journal.pgen.1006078 (PMC4882070; doi:10.1371/journal.pgen.1006078)
Supplement: S1 Table — P-values were calculated using Wald test by a linear mixed model correcting for sample relatedness. FCH, familial combined hyperlipidemia. (PDF) [file pgen.1006078.s007.pdf]

	FCH affected		FCH unaffected		
	n	**Mean ± SD**	***n***	**Mean ± SD**	***p*-value**
n (male/female)	258 (115/143)		907 (488/415)		$7.08x10^{-3}$
Smoking, *n* (%)	77 (30)		138 (15)		0.53
Age (year)	257	42.6±14.2	739	43.5±17.6	0.47
BMI (kg/m^2)	232	27.3±4.9	475	25.2±4.6	$1.04x10^{-6}$
Waist circumference (cm)	186	92±14	343	86±14	$1.58x10^{-6}$
Total cholesterol (mmol/l)	235	6.62±1.38	480	5.22±0.91	$2.53x10^{-58}$
LDL-C (mmol/l)	235	4.26±1.24	480	3.33±0.89	$1.86x10^{-29}$
Triglyceride (mmol/l)	235	2.55±2.05	480	1.13±0.50	$4.43x10^{-46}$
HDL-C (mmol/l)	231	1.23±0.40	471	1.40±0.42	$7.24x10^{-7}$
Apolipoprotein B (mg/dl)	214	125±32	450	88±22	$1.21x10^{-68}$
Non-HDL-C (mmol/l)	231	5.40±1.42	471	3.81±0.95	$1.13x10^{-67}$
