# Supplementary material for: The Contribution of GWAS Loci in Familial Dyslipidemias
Source: PLoS Genet. 2016 May 26;12(5):e1006078. doi: 10.1371/journal.pgen.1006078 (PMC4882070; doi:10.1371/journal.pgen.1006078)
Supplement: S2 Table — NFE, non-Finnish Europeans; FINRISK, The National FINRISK Study; FCH, familial combined hyperlipidemia. *Effect allele. †Global minor allele. ‡MAF in non-Finnish Europeans calculated from the public ExAC data set (version 0.3) for coding SNPs (those present in the ExAC data set), and from the public 1000 Genomes Phase 3 data set for non-coding SNPs (S1 Text). §MAF in the FINRISK cohort after excluding those with known diabetes or cancer. ∥Common: MAF > 5%, low-frequency: 0.5% < MAF ≤ 5%, rare: MAF ≤ 0.5%. ¶SNPs were selected based on OMIM entries for genes implicated in monogenic dyslipidemia, and recent genome-wide association studies (S1 Text). ♦Effect allele weight not estimated due to only one copy of minor allele present in FINRISK. (PDF) [file pgen.1006078.s008.pdf]

	Chr.	Gene	Associated lipid(s)	Minor allele[†]	Major allele	Effect allele weight(s)	Minor allele frequency					SNP frequency class[‖]	SNP source[¶]	SNP genotype class
							NFE[‡]	FINRISK[§]	FCH families	FCH affected	FCH probands			
rs586178	1	*LDLRAP1*	LDL-C	C*	G	-0.019	0.45	0.46	0.48	0.46	0.44	Common	Surakka	Imputed
rs12027135	1	*LDLRAP1*	LDL-C	T*	A	0.061	0.53	0.46	0.54	0.51	0.48	Common	Teslovich	Genotyped
rs12748152	1	*PIGV-NR0B2*	LDL-C, TG	T*	C	0.005, 0.025	0.075	0.079	0.11	0.15	0.17	Common	Willer	Genotyped
rs79598313	1	*PIGV-NR0B2*	LDL-C	T*	C	0.080	0.025	0.035	0.052	0.083	0.083	Low-frequency	Surakka	Imputed
rs2479409	1	*PCSK9*	LDL-C	G	A*	-0.029	0.32	0.29	0.26	0.24	0.23	Common	Teslovich	Genotyped
rs11591147	1	*PCSK9*	LDL-C	T*	G	-0.443	0.035	0.040	0.038	0.034	0.042	Low-frequency	Willer, Kuivenhoven	Genotyped
rs28362286	1	*PCSK9*	LDL-C, TG	A*	C	-0.894, -0.887	0	0	$4.2x10^{-6}$	$4.3x10^{-6}$	0	Rare	Kuivenhoven	Imputed
rs9988450	1	*DOCK7*	TG	T*	C	0.046	0.32	0.26	0.23	0.22	0.27	Common	Surakka	Imputed
rs2131925	1	*ANGPTL3*	TG	G	T*	0.089	0.32	0.26	0.23	0.22	0.27	Common	Teslovich	Genotyped
rs3850634	1	*ANGPTL3*	LDL-C	G*	T	-0.059	0.30	0.26	0.23	0.22	0.27	Common	Teslovich	Genotyped
rs10889348	1	*DOCK7*	LDL-C	T*	A	0.028	0.32	0.27	0.24	0.22	0.27	Common	Surakka	Imputed
rs11164654	1	*EVI5*	LDL-C	T	C*	0.028	0.19	0.22	0.23	0.26	0.25	Common	Surakka	Imputed
rs629301	1	*SORT1*	LDL-C	G	T*	-0.138	0.22	0.22	0.20	0.18	0.21	Common	Teslovich	Genotyped
rs646776	1	*CELSR2*	LDL-C	C	T*	0.277	0.22	0.21	0.20	0.18	0.21	Common	Surakka	Genotyped
rs267733	1	*ANXA9-CERS2*	LDL-C	G*	A	-0.023	0.15	0.14	0.13	0.13	0.12	Common	Willer	Genotyped
rs57830985	1	*LMNA*	LDL-C, TG	A*	G	-0.059, 0.004	0	$5.6x10^{-4}$	$1.3x10^{-5}$	0	0	Rare	Kuivenhoven	Imputed
rs340839	1	*PROX1*	TG	A*	G	0.002	0.50	0.38	0.44	0.42	0.46	Common	Surakka	Genotyped
rs2807834	1	*MOSC1*	LDL-C	T	G*	0.019	0.30	0.29	0.32	0.29	0.28	Common	Teslovich	Imputed
rs10864728	1	*GALNT2*	TG	G*	A	-0.130	0.61	0.43	0.56	0.57	0.53	Common	Surakka	Imputed
rs1321257	1	*GALNT2*	TG	A*	G	0.095	0.61	0.43	0.56	0.57	0.53	Common	Teslovich	Imputed
rs514230	1	*IRF2BP2*	LDL-C	A	T*	0.044	0.49	0.43	0.47	0.44	0.46	Common	Teslovich	Genotyped
rs4665710	2	*APOB*	TG	A	C*	0.119	0.22	0.26	0.26	0.27	0.26	Common	Surakka	Imputed
rs1042034	2	*APOB*	TG	C	T*	-0.076	0.21	0.26	0.26	0.27	0.26	Common	Teslovich	Genotyped
rs1367117	2	*APOB*	LDL-C	A*	G	0.122	0.32	0.28	0.29	0.30	0.32	Common	Teslovich	Genotyped
rs515135	2	*APOB*	LDL-C	T	C*	0.103	0.19	0.19	0.18	0.13	0.11	Common	Surakka	Genotyped
rs1260326	2	*GCKR*	TG	T	C*	-0.048	0.41	0.36	0.44	0.47	0.48	Common	Teslovich	Genotyped
rs11887534	2	*ABCG5/8*	LDL-C, TG	C*	G	-0.086, -0.017	0.077	0.091	0.068	0.062	0.052	Common	Kuivenhoven	Genotyped
rs4299376	2	*ABCG5/8*	LDL-C	G	T*	-0.057	0.33	0.21	0.21	0.24	0.32	Common	Teslovich	Genotyped
rs72875462	2	*ABCG8*	LDL-C	A*	C	-0.051	0.064	0.090	0.067	0.062	0.052	Common	Surakka	Imputed
rs137852990	2	*ABCG5/8*	LDL-C, TG	A*	G	0.212, 0.146	$1.0x10^{-4}$	$2.7x10^{-5}$	$1.3x10^{-4}$	$2.1x10^{-4}$	$7.3x10^{-5}$	Rare	Kuivenhoven	Imputed
rs137852987	2	*ABCG5/8*	LDL-C, TG	A*	G	0.043, -0.073	0.0015	0.0025	0.0042	0	0	Rare	Kuivenhoven	Genotyped
rs2710642	2	*EHBP1*	LDL-C	G	A*	0.026	0.38	0.39	0.44	0.44	0.45	Common	Willer	Imputed
rs2540948	2	*CEP68*	TG	C*	T	-0.011	0.37	0.32	0.33	0.31	0.32	Common	Surakka	Imputed
rs10490626	2	*INSIG2*	LDL-C	A*	G	-0.043	0.082	0.067	0.063	0.066	0.073	Common	Willer	Genotyped
rs2030746	2	*LOC84931*	LDL-C	T*	C	0.017	0.41	0.35	0.37	0.39	0.34	Common	Willer	Genotyped
rs10195252	2	*COBLL1*	TG	C*	T	-0.014	0.46	0.34	0.35	0.38	0.38	Common	Teslovich	Genotyped
rs140244541	2	*FAM117B*	LDL-C	A*	G	-0.021	0.16	0.13	0.11	0.12	0.14	Common	Surakka	Imputed
rs1250229	2	*FN1*	LDL-C	T	C*	0.024	0.25	0.20	0.21	0.21	0.24	Common	Willer	Genotyped
rs2943645	2	*IRS1*	TG	C	T*	0.015	0.37	0.37	0.37	0.39	0.40	Common	Teslovich	Imputed
rs11563251	2	*UGT1A1*	LDL-C	T*	C	0.033	0.12	0.098	0.14	0.16	0.16	Common	Willer	Imputed
rs2920503	3	*PPARG*	LDL-C	T*	C	0.012	0.28	0.32	0.34	0.37	0.35	Common	Surakka	Imputed

rs3856806	3	*PPARG*	LDL-C, TG	T*	C	0.035, 0.002	0.13	0.20	0.16	0.13	0.15	Common	Kuivenhoven	Genotyped
rs7640978	3	*CMTM6*	LDL-C	T*	C	-0.022	0.12	0.086	0.058	0.051	0.062	Common	Willer	Genotyped
rs17404153	3	*ACAD11*	LDL-C	T*	G	0.001	0.12	0.18	0.18	0.16	0.14	Common	Willer	Genotyped
rs34894639	3	*MSL2L1, PCCB*	TG	T*	C	0.015	0.26	0.18	0.17	0.18	0.15	Common	Surakka	Imputed
rs645040	3	*MSL2L1*	TG	G	T*	0.038	0.25	0.14	0.12	0.12	0.073	Common	Teslovich	Genotyped
rs6818397	4	*LRPAP1*	LDL-C	G*	T	0.014	0.57	0.27	0.65	0.65	0.61	Common	Willer	Genotyped
rs59950280	4	*LRPAP1*	TG	A*	G	0.003	0.31	0.23	0.31	0.30	0.30	Common	Surakka	Imputed
rs6831256	4	*LRPAP1*	TG	G*	A	0.016	0.40	0.35	0.42	0.42	0.43	Common	Willer	Genotyped
rs182616603	4	*MTHFD2L*	LDL-C	T*	C	0.280	0.0012	0.021	0.030	0.045	0.025	Low-frequency	Surakka	Imputed
rs2035403	4	*KLHL8*	TG	G*	A	0.004	0.38	0.40	0.41	0.40	0.39	Common	Surakka	Imputed
rs442177	4	*KLHL8*	TG	G	T*	0.017	0.40	0.48	0.49	0.49	0.51	Common	Teslovich	Genotyped
rs3816873	4	*MTTP*	LDL-C, TG	C*	T	-0.036, 0.004	0.26	0.19	0.19	0.22	0.21	Common	Kuivenhoven	Genotyped
rs9686661	5	*MAP3K1*	TG	T*	C	0.011	0.20	0.14	0.13	0.15	0.20	Common	Teslovich	Genotyped
rs3843482	5	*HMGCR*	LDL-C	G*	T	0.064	0.38	0.44	0.44	0.42	0.38	Common	Surakka	Imputed
rs12916	5	*HMGCR*	LDL-C	C*	T	0.025	0.40	0.46	0.45	0.45	0.41	Common	Teslovich	Genotyped
rs4530754	5	*CSNK1G3*	LDL-C	G	A*	0.034	0.39	0.50	0.48	0.52	0.48	Common	Willer	Genotyped
rs6882076	5	*TIMD4*	LDL-C	T	C*	0.082	0.36	0.33	0.34	0.34	0.38	Common	Teslovich	Genotyped
rs1553318	5	*TIMD4*	LDL-C, TG	G	C*	-0.035, 0.018	0.36	0.32	0.35	0.35	0.36	Common	Teslovich, Surakka	Imputed
rs3757354	6	*MYLIP*	LDL-C	T*	C	-0.026	0.20	0.26	0.28	0.29	0.32	Common	Teslovich	Genotyped
rs1800562	6	*HFE*	LDL-C	A*	G	-0.036	0.051	0.038	0.048	0.047	0.021	Low-frequency	Teslovich	Genotyped
rs2247056	6	*HLA*-area	TG	T	C*	0.022	0.21	0.23	0.24	0.23	0.21	Common	Teslovich	Genotyped
rs419132	6	*HLA*-area	TG	G*	A	0.006	0.30	0.16	0.16	0.15	0.14	Common	Surakka	Imputed
rs3177928	6	*HLA*-area	LDL-C	A*	G	0.038	0.16	0.21	0.22	0.24	0.22	Common	Teslovich	Genotyped
rs114067101	6	*HLA*-area	LDL-C	G*	A	0.022	0.058	0.0077	0.046	0.050	0.037	Low-frequency	Surakka	Imputed
rs998584	6	*VEGFA*	TG	A*	C	0.037	0.50	0.49	0.52	0.57	0.61	Common	Willer	Genotyped
rs1358980	6	*VEGFA*	TG	T*	C	-0.016	0.49	0.47	0.47	0.50	0.56	Common	Surakka	Genotyped
rs11153594	6	*FRK*	LDL-C	T*	C	-0.024	0.41	0.40	0.41	0.41	0.44	Common	Teslovich	Imputed
rs719726	6	*RSPO3*	TG	C	T*	0.006	0.48	0.44	0.42	0.40	0.40	Common	Willer	Imputed
rs72959041	6	*RSPO3*	TG	A*	G	0.031	0.048	0.062	0.088	0.12	0.12	Common	Surakka	Imputed
rs17585887	6	*CITED2*	TG	C*	T	-0.016	0.60	0.50	0.51	0.54	0.52	Common	Surakka	Genotyped
rs2297374	6	*LPA*	LDL-C	T*	C	-0.013	0.40	0.34	0.36	0.33	0.36	Common	Willer	Imputed
rs1564348	6	*LPA*	LDL-C	C*	T	0.021	0.17	0.14	0.17	0.18	0.15	Common	Teslovich	Genotyped
rs7759633	6	*LPA*	TG	A*	G	-0.009	0.14	0.093	0.11	0.12	0.10	Common	Surakka	Imputed
rs186696265	6	*LPA*	LDL-C	T*	C	0.202	0.011	0.013	0.035	0.025	0.020	Low-frequency	Surakka	Imputed
rs56130071	7	*DNAH11*	LDL-C	C*	G	0.003	0.19	0.21	0.24	0.24	0.19	Common	Surakka	Imputed
rs12670798	7	*DNAH11*	LDL-C	C*	T	0.034	0.22	0.24	0.27	0.28	0.22	Common	Teslovich	Genotyped
rs4722551	7	*MIR148A*	LDL-C	C*	T	0.048	0.18	0.23	0.24	0.21	0.22	Common	Willer	Genotyped
rs4719841	7	*MIR148A*	TG	G*	A	-0.001	0.40	0.37	0.37	0.40	0.38	Common	Willer	Genotyped
rs41279633	7	*NPC1L1*	LDL-C	T*	G	0.028	0.17	0.21	0.23	0.26	0.29	Common	Surakka	Imputed
rs2073547	7	*NPC1L1*	LDL-C	G*	A	-0.002	0.17	0.34	0.37	0.38	0.41	Common	Willer	Imputed
rs217386	7	*NPC1L1*	LDL-C	A*	G	-0.031	0.43	0.32	0.30	0.27	0.26	Common	Teslovich	Imputed
rs13238203	7	*TYW1B*	TG	T*	C	-0.027	0.029	0.013	0	0	0	Low-frequency	Teslovich	Genotyped
rs7811265	7	*MLXIPL*	TG	G*	A	0.027	0.19	0.17	0.18	0.15	0.16	Common	Teslovich	Imputed
rs9638182	7	*MLXIPL*	TG	G*	T	-0.076	0.19	0.17	0.18	0.15	0.16	Common	Surakka	Imputed

rs2255811	7	*GPR85*	TG	G	A*	-0.001	0.28	0.23	0.22	0.23	0.26	Common	Surakka	Imputed
rs38855	7	*MET*	TG	G*	A	0.002	0.48	0.46	0.45	0.42	0.47	Common	Willer	Genotyped
rs2169387	8	*PPP1R3B*	LDL-C	A	G*	0.005	0.083	0.15	0.12	0.10	0.14	Common	Surakka	Imputed
rs2126259	8	*PPP1R3B*	LDL-C	T	C*	0.038	0.084	0.16	0.13	0.11	0.14	Common	Teslovich	Genotyped
rs11776767	8	*PINX1*	TG	C*	G	0.005	0.41	0.31	0.31	0.30	0.29	Common	Teslovich	Genotyped
rs1495743	8	*NAT2*	TG	G	C*	-0.008	0.24	0.25	0.25	0.22	0.19	Common	Teslovich	Imputed
rs1801177	8	*LPL*	LDL-C, TG	A*	G	0.097, 0.009	0.015	0.0018	0.0056	0.011	0.010	Rare	Kuivenhoven	Genotyped
rs268	8	*LPL*	LDL-C, TG	G*	A	0.032, 0.109	0.020	0.022	0.028	0.032	0.021	Low-frequency	Kuivenhoven	Genotyped
rs326	8	*LPL*	LDL-C, TG	G*	A	0.205, 0.014	0.34	0.26	0.22	0.19	0.15	Common	Kuivenhoven	Genotyped
rs328	8	*LPL*	LDL-C, TG	G*	C	-0.025, -0.005	0.10	0.088	0.071	0.056	0.031	Common	Kuivenhoven	Genotyped
rs13702	8	*LPL*	LDL-C, TG	C*	T	-0.217, -0.039	0.34	0.26	0.21	0.18	0.15	Common	Kuivenhoven	Genotyped
rs12678919	8	*LPL*	TG	G*	A	-0.031	0.13	0.086	0.070	0.056	0.031	Common	Teslovich	Genotyped
rs115849089	8	*LPL*	TG	A*	G	-0.037	0.15	0.10	0.082	0.067	0.053	Common	Surakka	Imputed
rs10102164	8	*SOX17*	LDL-C	A*	G	0.041	0.17	0.24	0.28	0.29	0.28	Common	Willer	Genotyped
rs1030431	8	*CYP7A1*	LDL-C	A	G*	-0.000	0.35	0.31	0.31	0.30	0.35	Common	Teslovich	Imputed
rs9297994	8	*CYP7A1*	LDL-C	G	A*	-0.039	0.36	0.38	0.39	0.39	0.39	Common	Surakka	Imputed
rs2954022	8	*TRIB1*	LDL-C	A*	C	-0.010	0.45	0.47	0.45	0.44	0.45	Common	Teslovich	Imputed
rs2954029	8	*TRIB1*	TG	T*	A	-0.040	0.45	0.46	0.44	0.43	0.44	Common	Teslovich	Genotyped
rs112875651	8	*TRIB1*	LDL-C	A*	G	-0.036	0.38	0.39	0.36	0.34	0.36	Common	Surakka	Imputed
rs11136341	8	*PLEC1*	LDL-C	G*	A	-0.021	0.41	0.37	0.40	0.41	0.43	Common	Teslovich	Genotyped
rs11784833	8	*GRINA, PLEC1*	LDL-C	C*	T	0.048	0.40	0.39	0.43	0.45	0.47	Common	Surakka	Imputed
rs3780181	9	*VLDLR*	LDL-C	G*	A	-0.047	0.056	0.053	0.053	0.060	0.052	Common	Willer	Genotyped
rs2230806	9	*ABCA1*	LDL-C, TG	T*	C	0.019, -0.006	0.27	0.22	0.18	0.19	0.18	Common	Kuivenhoven	Genotyped
rs3890182	9	*ABCA1*	LDL-C, TG	A*	G	-0.024, 0.000	0.12	0.11	0.098	0.094	0.073	Common	Kuivenhoven	Genotyped
rs550057	9	*ABO*	LDL-C	T*	C	0.049	0.28	0.31	0.29	0.30	0.31	Common	Surakka	Imputed
rs649129	9	*ABO*	LDL-C	T*	C	0.034	0.22	0.17	0.18	0.19	0.20	Common	Teslovich	Imputed
rs116807569	9	*AGPAT2*	LDL-C, TG	C*	T	◊	0	0	$7.0x10^{-6}$	$6.4x10^{-6}$	$3.1x10^{-5}$	Rare	Kuivenhoven	Imputed
rs1832007	10	*AKR1C4*	TG	G*	A	-0.009	0.12	0.13	0.14	0.13	0.10	Common	Willer	Genotyped
rs10822145	10	*JMJD1C*	TG	T*	C	-0.019	0.51	0.48	0.50	0.46	0.44	Common	Surakka	Imputed
rs10761731	10	*JMJD1C*	TG	T*	A	0.000	0.45	0.38	0.39	0.34	0.31	Common	Teslovich	Genotyped
rs2068888	10	*CYP26A1*	TG	A*	G	-0.018	0.49	0.47	0.48	0.49	0.46	Common	Teslovich	Genotyped
rs1129555	10	*GPAM*	LDL-C	A	G*	-0.036	0.31	0.33	0.29	0.29	0.28	Common	Teslovich	Genotyped
rs174529	11	*FADS1*	TG	C*	T	-0.007	0.36	0.42	0.42	0.43	0.45	Common	Surakka	Imputed
rs174546	11	*FADS1-2-3*	TG	T*	C	0.031	0.32	0.42	0.41	0.42	0.45	Common	Teslovich	Genotyped
rs174551	11	*FADS1*	LDL-C	C*	T	0.002	0.32	0.39	0.39	0.39	0.42	Common	Surakka	Imputed
rs174583	11	*FADS1-2-3*	LDL-C	T*	C	-0.068	0.34	0.42	0.40	0.41	0.44	Common	Teslovich	Genotyped
rs964184	11	*APOA1-C3-A4-A5*	LDL-C, TG	G	C*	0.075, 0.048	0.16	0.14	0.18	0.23	0.23	Common	Teslovich	Genotyped
rs2266788	11	*APOA5*	LDL-C, TG	G	A*	-0.166, -0.109	0.095	0.084	0.094	0.12	0.094	Common	Kuivenhoven	Genotyped
rs2075291	11	*APOA5*	LDL-C, TG	A*	C	0.030, -0.100	$3.2x10^{-4}$	$6.4x10^{-4}$	0	0	0	Rare	Kuivenhoven	Genotyped
rs2072560	11	*APOA5*	LDL-C, TG	T	C*	-0.148, 0.035	0.067	0.080	0.094	0.12	0.094	Common	Kuivenhoven	Imputed
rs3135506	11	*APOA5*	LDL-C, TG	C*	G	0.165, 0.186	0.079	0.061	0.088	0.11	0.14	Common	Kuivenhoven	Genotyped
rs651821	11	*APOA5*	LDL-C, TG	C	T*	0.124, 0.145	0.071	0.081	0.094	0.12	0.094	Common	Kuivenhoven	Genotyped
rs662799	11	*APOA5*	LDL-C, TG	G	A*	0.024, -0.277	0.088	0.080	0.094	0.12	0.094	Common	Kuivenhoven	Imputed
rs121912720	11	*APOA1*	LDL-C, TG	T*	G	-0.064, -0.107	0	0.0020	0.0014	0	0	Rare	Kuivenhoven	Genotyped
rs59379014	11	*ST3GAL4*	LDL-C	T*	C	0.043	0.063	0.077	0.069	0.070	0.062	Common	Willer	Imputed

rs11220462	11	*ST3GAL4*	LDL-C	A*	G	0.043	0.12	0.19	0.18	0.18	0.15	Common	Teslovich	Genotyped
rs4307732	11	*ST3GAL4*	LDL-C	A*	G	-0.019	0.094	0.16	0.15	0.15	0.14	Common	Surakka	Imputed
rs11613352	12	*LRP1*	TG	T*	C	0.085	0.20	0.23	0.23	0.25	0.19	Common	Teslovich	Genotyped
rs61352607	12	*LRP1*	TG	T*	G	-0.092	0.20	0.23	0.23	0.25	0.19	Common	Surakka	Imputed
rs11065987	12	*BRAP*	LDL-C	G*	A	-0.019	0.44	0.38	0.37	0.38	0.40	Common	Teslovich	Genotyped
rs1169288	12	*HNF1A*	LDL-C	C*	A	0.024	0.35	0.39	0.37	0.40	0.48	Common	Kuivenhoven, Teslovich	Genotyped
rs137853247	12	*HNF1A*	LDL-C, TG	A*	G	0.357, 0.121	0.0010	$4.0x10^{-4}$	$9.0x10^{-5}$	$8.3x10^{-5}$	0	Rare	Kuivenhoven	Imputed
rs1169314	12	*HNF1A*	LDL-C	G*	A	0.024	0.33	0.31	0.31	0.33	0.40	Common	Surakka	Imputed
rs12310367	12	*ZNF664*	TG	G*	A	-0.008	0.38	0.28	0.29	0.29	0.23	Common	Teslovich	Imputed
rs4942486	13	*BRCA2*	LDL-C	T	C*	-0.010	0.49	0.43	0.39	0.37	0.33	Common	Willer	Genotyped
rs11621792	14	*NYNRIN*	LDL-C	T*	C	-0.006	0.49	0.36	0.35	0.36	0.42	Common	Surakka	Imputed
rs2332328	14	*NYNRIN*	LDL-C	T*	C	0.028	0.51	0.38	0.39	0.40	0.44	Common	Teslovich	Imputed
rs2412710	15	*CAPN3*	TG	A*	G	-0.046	0.035	0.0026	0.0035	0.0043	0.010	Rare	Teslovich	Genotyped
rs62020698	15	*UBR1, CAPN3*	TG	T*	C	-0.004	0.12	0.025	0.048	0.065	0.086	Low-frequency	Surakka	Imputed
rs2929282	15	*FRMD5*	TG	T*	A	-0.016	0.051	0.049	0.049	0.060	0.042	Low-frequency	Teslovich	Genotyped
rs138570705	15	*FRMD5*	TG	A*	G	-0.016	0.033	0.018	0.022	0.028	0.021	Low-frequency	Surakka	Imputed
rs1077835	15	*LIPC*	TG	G*	A	0.048	0.22	0.27	0.26	0.27	0.28	Common	Surakka	Imputed
rs261342	15	*LIPC*	TG	G	C*	0.007	0.22	0.23	0.23	0.23	0.22	Common	Teslovich	Imputed
rs12594375	15	*LIPC*	LDL-C, TG	A*	G	0.027, 0.091	0.012	0.019	0.018	0.019	0.021	Low-frequency	Kuivenhoven	Imputed
rs8023503	15	*LIPC*	LDL-C, TG	T*	C	-0.060, -0.075	0.014	0.019	0.018	0.019	0.021	Low-frequency	Kuivenhoven	Imputed
rs121912502	15	*LIPC*	LDL-C, TG	T*	C	0.574, 0.042	0.0020	$2.7x10^{-5}$	$6.3x10^{-6}$	$2.2x10^{-6}$	0	Rare	Kuivenhoven	Imputed
rs28933094	15	*LIPC*	LDL-C, TG	T*	C	0.020, 0.114	0.0033	0.016	0.034	0.041	0.031	Low-frequency	Kuivenhoven	Genotyped
rs3198697	16	*PDXDC1*	TG	T*	C	-0.007	0.42	0.41	0.42	0.42	0.46	Common	Willer	Imputed
rs11649653	16	*CTF1*	TG	G*	C	-0.004	0.40	0.42	0.43	0.37	0.40	Common	Teslovich	Genotyped
rs1121980	16	*FTO*	TG	A*	G	0.019	0.45	0.43	0.38	0.39	0.34	Common	Willer	Genotyped
rs247616	16	*CETP*	LDL-C	T*	C	-0.077	0.30	0.28	0.30	0.29	0.32	Common	Teslovich	Genotyped
rs247617	16	*CETP*	LDL-C	A*	C	0.072	0.30	0.28	0.30	0.29	0.32	Common	Surakka	Genotyped
rs183130	16	*CETP*	LDL-C, TG	T*	C	-0.041, -0.248	0.30	0.28	0.30	0.29	0.32	Common	Kuivenhoven	Genotyped
rs821840	16	*CETP*	TG	G*	A	0.240	0.29	0.28	0.30	0.29	0.32	Common	Surakka	Imputed
rs7205804	16	*CETP*	TG	A*	G	-0.001	0.43	0.42	0.45	0.44	0.44	Common	Teslovich	Genotyped
rs5882	16	*CETP*	LDL-C, TG	G	A*	0.006, 0.009	0.32	0.38	0.40	0.42	0.45	Common	Kuivenhoven	Genotyped
rs2303790	16	*CETP*	LDL-C, TG	G*	A	-0.381, 0.012	$1.5x10^{-4}$	$2.4x10^{-4}$	$7.0x10^{-4}$	0	0	Rare	Kuivenhoven	Genotyped
rs11648003	16	*HPR*	LDL-C	G*	A	0.027	0.23	0.22	0.22	0.24	0.19	Common	Surakka	Imputed
rs2000999	16	*HPR*	LDL-C	A*	G	0.036	0.19	0.19	0.19	0.21	0.16	Common	Teslovich	Genotyped
rs193042029	17	*TM4SF5*	TG	G*	T	-0.067	0.0037	0.029	0.029	0.024	0.031	Low-frequency	Surakka	Imputed
rs55714927	17	*DLG4*	LDL-C	T*	C	-0.020	0.19	0.25	0.30	0.31	0.30	Common	Surakka	Imputed
rs314253	17	*DLG4*	LDL-C	C*	T	-0.016	0.32	0.41	0.45	0.47	0.48	Common	Willer	Genotyped
rs80356487	17	*G6PC*	LDL-C, TG	T*	C	2.165, 0.440	$4.4x10^{-4}$	0	$5.5x10^{-5}$	$5.6x10^{-5}$	0	Rare	Kuivenhoven	Imputed
rs77697917	17	*DUSP3*	TG	T*	C	0.038	0.041	0.028	0.037	0.059	0.062	Low-frequency	Surakka	Imputed
rs8077889	17	*MPP3*	TG	C*	A	0.027	0.26	0.21	0.23	0.23	0.22	Common	Willer	Genotyped
rs72836561	17	*CD300LG*	TG	T*	C	0.050	0.029	0.028	0.040	0.064	0.073	Low-frequency	Surakka	Genotyped
rs7225700	17	*OSBPL7*	LDL-C	T	C*	-0.003	0.34	0.32	0.30	0.27	0.27	Common	Teslovich	Genotyped
rs7206971	17	*OSBPL7*	LDL-C	A*	G	0.025	0.48	0.49	0.51	0.55	0.53	Common	Surakka	Genotyped
rs1801689	17	*APOH-PRXCA*	LDL-C	C*	A	0.062	0.033	0.0080	0.011	0.011	0.010	Low-frequency	Willer	Genotyped

rs77542162	17	*ABCA6*	LDL-C	G*	A	0.212	0.017	0.0053	0.0077	0.0021	0	Low-frequency	Surakka	Genotyped
rs79588679	18	*GATA6*	LDL-C	T*	C	-0.012	0.16	0.17	0.16	0.16	0.15	Common	Surakka	Imputed
rs1799816	19	*INSR*	LDL-C, TG	T*	C	0.015, 0.045	0.0093	0.0063	0.0035	0.0043	0	Low-frequency	Kuivenhoven	Genotyped
rs7248104	19	*INSR*	TG	A*	G	-0.006	0.44	0.44	0.41	0.39	0.42	Common	Willer	Genotyped
rs116843064	19	*ANGPTL4*	TG	A*	G	-0.099	0.050	0.026	0.015	0.0043	0	Low-frequency	Surakka	Genotyped
rs112374545	19	*LDLR*	LDL-C	T*	C	-0.066	0.12	0.11	0.094	0.098	0.094	Common	Surakka	Imputed
rs6511720	19	*LDLR*	LDL-C	T*	G	-0.171	0.11	0.11	0.095	0.098	0.094	Common	Teslovich	Genotyped
rs58542926	19	*TM6SF2*	LDL-C, TG	T*	C	-0.119, 0.067	0.081	0.059	0.052	0.053	0.083	Common	Surakka	Genotyped
rs187429064	19	*TM6SF2*	LDL-C, TG	G*	A	-0.119, -0.064	0.013	0.052	0.046	0.037	0.042	Common	Surakka	Imputed
rs10401969	19	*CILP2*	LDL-C, TG	C*	T	-0.075, -0.144	0.073	0.059	0.052	0.053	0.083	Common	Teslovich	Genotyped
rs73001065	19	*CILP2*	LDL-C	C*	G	0.065	0.064	0.057	0.052	0.053	0.082	Common	Surakka	Imputed
rs731839	19	*PEPD*	TG	G	A*	-0.005	0.33	0.35	0.33	0.31	0.34	Common	Willer	Genotyped
rs405509	19	*APOE*	LDL-C, TG	T	G*	0.021, -0.015	0.50	0.45	0.43	0.42	0.42	Common	Kuivenhoven	Imputed
rs121918392	19	*APOE*	LDL-C, TG	A*	G	-0.670, -0.210	0	$1.9x10^{-4}$	$7.0x10^{-4}$	0	0	Rare	Kuivenhoven	Imputed
rs201672011	19	*APOE*	LDL-C, TG	A*	G	0.662, 0.389	$1.1x10^{-4}$	$2.7x10^{-5}$	0	0	0	Rare	Kuivenhoven	Imputed
rs769452	19	*APOE*	LDL-C, TG	C*	T	0.019, -0.001	0.0036	0.0056	0.0056	0.0064	0.010	Low-frequency	Kuivenhoven	Genotyped
rs429358	19	*APOE*	LDL-C, TG	C*	T	0.156, 0.002	0.21	0.19	0.23	0.25	0.21	Common	Kuivenhoven	Imputed
rs769455	19	*APOE*	LDL-C, TG	T*	C	-0.274, -0.254	0	$2.7x10^{-5}$	0.0021	0.0043	0.010	Rare	Kuivenhoven	Imputed
rs7412	19	*APOE*	LDL-C	T*	C	-0.531	0.11	0.047	0.074	0.081	0.10	Low-frequency	Kuivenhoven, Willer	Genotyped
rs140808909	19	*APOE*	LDL-C, TG	A*	G	6.423, -0.160	0	0	$8.4x10^{-6}$	$6.4x10^{-6}$	0	Rare	Kuivenhoven	Imputed
rs190853081	19	*APOE*	LDL-C, TG	A*	G	1.190, 2.834	0	0	$8.4x10^{-6}$	$6.4x10^{-6}$	0	Rare	Kuivenhoven	Imputed
rs439401	19	*APOE-C1-C2*	TG	T	C*	0.044	0.40	0.28	0.24	0.20	0.24	Common	Teslovich	Genotyped
rs438811	19	*APOE*	TG	T*	C	0.022	0.21	0.24	0.30	0.33	0.31	Common	Surakka	Imputed
rs4420638	19	*APOE-C1-C2*	LDL-C	G*	A	0.030	0.18	0.28	0.31	0.34	0.28	Common	Teslovich	Genotyped
rs120074114	19	*APOC2*	LDL-C, TG	C*	A	0.092, -0.120	0.0014	$2.9x10^{-4}$	$4.2x10^{-6}$	0	0	Rare	Kuivenhoven	Imputed
rs117492019	19	*ZNF274*	LDL-C	T*	G	-0.021	0.20	0.16	0.14	0.13	0.083	Common	Surakka	Imputed
rs364585	20	*SPTLC3*	LDL-C	A	G*	0.014	0.37	0.34	0.34	0.33	0.31	Common	Willer	Genotyped
rs2618568	20	*SNX5*	LDL-C	A*	C	-0.045	0.56	0.37	0.63	0.62	0.70	Common	Surakka	Imputed
rs2328223	20	*SNX5*	LDL-C	C*	A	-0.022	0.24	0.21	0.24	0.25	0.19	Common	Willer	Genotyped
rs6120974	20	*ERGIC3*	TG	C*	T	-0.005	0.16	0.058	0.053	0.037	0.044	Common	Surakka	Imputed
rs2902941	20	*MAFB*	LDL-C	G*	A	-0.012	0.31	0.26	0.26	0.25	0.25	Common	Teslovich	Imputed
rs1883711	20	*MAFB*	LDL-C	C*	G	0.136	0.036	0.053	0.067	0.089	0.086	Common	Surakka	Imputed
rs909802	20	*TOP1*	LDL-C	C	T*	0.023	0.55	0.46	0.46	0.43	0.48	Common	Teslovich	Imputed
rs4810479	20	*PLTP*	TG	C	T*	-0.005	0.28	0.26	0.27	0.32	0.31	Common	Teslovich	Genotyped
rs6073958	20	*PLTP*	TG	C*	T	0.023	0.22	0.18	0.19	0.22	0.24	Common	Surakka	Imputed
rs5763662	22	*MTMR3*	LDL-C	T*	C	0.053	0.024	0.031	0.023	0.024	0.010	Low-frequency	Willer	Genotyped
rs5756931	22	*PLA2G6*	TG	C*	T	-0.003	0.41	0.39	0.37	0.38	0.35	Common	Teslovich	Genotyped
rs4253776	22	*PPARA*	LDL-C	G*	A	0.046	0.12	0.079	0.063	0.060	0.083	Common	Willer	Genotyped
