# Supplementary material for: The Contribution of GWAS Loci in Familial Dyslipidemias
Source: PLoS Genet. 2016 May 26;12(5):e1006078. doi: 10.1371/journal.pgen.1006078 (PMC4882070; doi:10.1371/journal.pgen.1006078)
Supplement: S3 Table — Under the null hypothesis of no enrichment, we established the null distribution for enrichment testing by calculating the enrichment statistic (enrichment ratio) for all variants with effect allele (minor allele in the FINRISK cohort) frequency > 0.001% across the genome excluding the loci of the 212 SNPs. Enrichment ratios were calculated for each of the 212 SNPs if at least one carrier was identified (194 SNPs for affected individuals, 191 SNPs for probands), and one-tailed p-values were estimated from the null distribution. Enrichment ratios are presented for the affected individuals. The carrier counts were calculated from all genotyped FCH family members who passed exclusion criteria and did not have diabetes or other relevant confounders (n = 661), and did not account for non-independence within family. The + and—symbols denote elevating and lowering effects on the lipid in question, respectively. FCH, familial combined hyperlipidemia; FINRISK, The National FINRISK Study. J.A.K, J. A. Kuivenhoven; C.J.W., C. J. Willer; T.M.T., T. M. Teslovich; I.S., I. Surakka (S1 Text). †Effect allele frequencies in non-Finnish Europeans were calculated from the public ExAC data set (version 0.3) for coding SNPs (those present in the ExAC data set), and from the public 1000 Genomes Phase 3 data set for non-coding SNPs (S1 Text). ‡Effect allele frequencies in the FINRISK cohort after excluding those with known diabetes or cancer. §Enrichment ratio is the ratio of the effect allele frequencies in affected individuals (n = 234) to the Finnish population (n = 18,715). (PDF) [file pgen.1006078.s009.pdf]

						Effect Allele Frequency							
	**Gene**	**Effect allele**	**Source**	**Effect on LDL-C**	**Effect on TG**	**NFE† (%)**	**FINRISK‡ (%)**	**FCH Affected (%)**	**Enrichment Ratio§**	***P*-value in affected individuals**	***P*-value in probands**	**Hetero-zygotes (Affected)**	**Homo-zygotes (Affected)**
rs1260326	GCKR	T	T.M.T.	+	+	41	36	47	1.3	0.001	0.0097	317 (125)	132 (47)
rs12748152	PIGV-NR0B2	T	C.J.W.	+	+	7.5	7.9	15	1.9	0.0015	0.0076	125 (57)	10 (7)
rs62020698	UBR1, CAPN3	T	I.S.	+	-	12	2.7	6.5	2.4	0.0017	0.00099	52 (24)	1 (1)
rs79598313	PIGV-NR0B2	T	I.S.	+	+	2.5	3.5	8.3	2.4	0.002	0.026	59 (29)	6 (5)
rs11563251	UGT1A1	T	C.J.W.	+	+	12	10	16	1.6	0.0032	0.042	129 (54)	13 (6)
rs116843064	ANGPTL4	A	I.S.	+	-	5	2.7	0.43	0.16	0.0032		21 (2)	0 (0)
rs964184	APOA1-C3-A4-A5	G	T.M.T.	+	+	16	14	23	1.6	0.004	0.04	208 (91)	18 (8)
rs3135506	APOA5	C	J.A.K.	+	+	7.9	6.2	11	1.8	0.0041	0.0049	105 (43)	6 (5)
rs72959041	RSPO3	A	I.S.	+	+	4.8	6.6	12	1.8	0.0044	0.034	94 (45)	5 (3)
rs13702	LPL	C	J.A.K.	-	-	34	26	18	0.69	0.0044	0.004	223 (72)	27 (6)
rs72836561	CD300LG	T	I.S.	-	+	2.9	2.9	6.4	2.2	0.0048	0.015	51 (28)	1 (1)
rs438811	APOE	T	I.S.	+	+	21	24	33	1.4	0.0051	0.1	296 (110)	53 (22)
rs439401	APOE-C1-C2	T	T.M.T.	-	-	40	28	20	0.71	0.0067	0.34	231 (68)	44 (13)
rs326	LPL	G	J.A.K.	-	-	34	26	19	0.71	0.0082	0.0038	227 (73)	29 (7)
rs77697917	DUSP3	T	I.S.	-	+	4.1	2.8	5.9	2.1	0.01	0.045	44 (24)	1 (1)
rs6818397	LRPAP1	T	C.J.W.	-	+	43	27	35	1.3	0.013	0.018	284 (104)	88 (30)
rs28933094	LIPC	T	J.A.K.	+	+	0.33	1.6	4.1	2.6	0.015	0.22	45 (19)	0 (0)
rs515135	APOB	T	I.S.	-	-	19	19	13	0.67	0.026	0.06	182 (50)	21 (5)
rs3856806	PPARG	T	J.A.K.	+	-	13	20	13	0.68	0.028	0.22	155 (53)	26 (5)
rs998584	VEGFA	A	C.J.W.	+	+	50	49	57	1.2	0.03	0.015	332 (125)	186 (70)
rs2126259	PPP1R3B	T	T.M.T.	-	-	8.4	16	11	0.68	0.032	0.46	144 (48)	8 (2)
rs2169387	PPP1R3B	A	I.S.	-	+	8.3	15	10	0.69	0.033	0.62	136 (45)	8 (2)
rs115849089	LPL	A	I.S.	-	-	15	11	6.7	0.63	0.038	0.047	95 (29)	4 (1)
rs59950280	LRPAP1	A	I.S.	+	+	31	23	30	1.3	0.039	0.14	270 (92)	65 (24)
rs6831256	LRPAP1	G	C.J.W.	+	+	40	35	42	1.2	0.043	0.11	327 (113)	116 (41)

rs1801177	LPL	A	J.A.K.	+	+	1.5	0.19	1.1	5.6	0.05	0.22	8 (5)	0 (0)
rs429358	APOE	C	J.A.K.	+	+	21	19	25	1.3	0.051	0.64	237 (93)	30 (11)
rs10102164	SOX17	A	C.J.W.	+	+	17	24	29	1.2	0.061	0.32	272 (112)	48 (13)
rs41279633	NPC1L1	T	I.S.	+	-	17	21	26	1.2	0.062	0.054	229 (88)	34 (17)
rs4420638	APOE-C1-C2	G	T.M.T.	+	+	18	28	34	1.2	0.064	0.99	279 (114)	64 (23)
rs7412	APOE	T	J.A.K., C.J.W.	-	+	11	4.7	8.1	1.7	0.074	0.044	96 (34)	3 (2)
rs4810479	PLTP	C	T.M.T.	-	+	28	26	32	1.2	0.075	0.28	280 (113)	44 (18)
rs182616603	MTHFD2L	T	I.S.	+	+	0.12	2.4	4.5	1.9	0.087	0.95	27 (15)	0 (0)
rs7225700	OSBPL7	T	T.M.T.	-	+	34	32	27	0.83	0.089	0.27	296 (113)	48 (6)
rs6120974	ERGIC3	C	I.S.	-	+	16	6.2	3.7	0.6	0.09	0.41	57 (15)	3 (0)
rs7640978	CMTM6	T	C.J.W.	-	-	12	8.6	5.1	0.6	0.092	0.45	75 (24)	0 (0)
rs1883711	MAFB	C	I.S.	+	+	3.6	6.2	8.9	1.4	0.099	0.3	71 (37)	2 (1)
rs11784833	GRINA,PLEC1	C	I.S.	+	+	40	39	45	1.2	0.1	0.12	328 (114)	120 (47)
rs7206971	OSBPL7	G	I.S.	+	+	52	49	55	1.1	0.1	0.41	352 (134)	148 (39)
rs11649653	CTF1	G	T.M.T.	-	-	40	42	37	0.87	0.1	0.59	319 (102)	121 (35)
rs662799	APOA5	G	J.A.K.	+	+	8.8	8.1	12	1.4	0.11	0.65	117 (52)	5 (1)
rs2072560	APOA5	T	J.A.K.	+	+	6.7	8.1	12	1.4	0.11	0.66	117 (52)	5 (1)
rs651821	APOA5	C	J.A.K.	+	+	7.1	8.1	12	1.4	0.11	0.66	117 (52)	5 (1)
rs314253	DLG4	C	C.J.W.	-	-	32	41	47	1.1	0.11	0.18	368 (136)	112 (41)
rs4942486	BRCA2	T	C.J.W.	+	+	49	43	37	0.87	0.11	0.059	319 (102)	98 (36)
rs328	LPL	G	J.A.K.	-	-	10	8.8	5.6	0.63	0.12	0.052	89 (26)	2 (0)
rs112875651	TRIB1	A	I.S.	-	-	38	39	34	0.86	0.13	0.5	304 (100)	83 (27)
rs2479409	PCSK9	G	T.M.T.	+	+	32	29	24	0.83	0.13	0.2	270 (91)	38 (11)
rs646776	CELSR2	C	I.S.	-	+	22	21	18	0.82	0.14	0.88	212 (70)	23 (6)
rs629301	SORT1	G	T.M.T.	-	+	22	22	18	0.81	0.14	0.86	206 (70)	29 (6)
rs2266788	APOA5	G	J.A.K.	+	+	9.5	8.4	12	1.4	0.15	0.74	117 (52)	5 (1)
rs6073958	PLTP	C	I.S.	-	+	22	18	22	1.2	0.15	0.15	211 (93)	17 (5)
rs55714927	DLG4	T	I.S.	-	-	19	26	31	1.2	0.15	0.43	243 (86)	51 (19)

rs12678919	LPL	G	T.M.T.	-	-	13	8.6	5.6	0.65	0.15	0.058	88 (26)	2 (0)
rs1564348	LPA	C	T.M.T.	+	-	17	14	18	1.3	0.16	0.85	193 (76)	18 (3)
rs10889348	DOCK7	T	I.S.	-	-	32	27	22	0.84	0.16	0.92	237 (86)	33 (9)
rs2073547	NPC1L1	G	C.J.W.	+	-	17	34	38	1.1	0.17	0.16	303 (103)	92 (38)
rs7248104	INSR	A	C.J.W.	-	-	44	44	39	0.89	0.17	0.66	336 (109)	101 (37)
rs187429064	TM6SF2	G	I.S.	-	-	1.3	5.6	3.7	0.67	0.17	0.49	46 (15)	2 (0)
rs2328223	SNX5	C	C.J.W.	+	+	24	21	25	1.2	0.18	0.51	251 (86)	38 (16)
rs2131925	ANGPTL3	G	T.M.T.	-	-	32	26	22	0.85	0.18	0.77	240 (85)	30 (9)
rs9988450	DOCK7	T	I.S.	-	-	32	26	22	0.85	0.18	0.79	240 (85)	30 (9)
rs2710642	EHBP1	G	C.J.W.	-	+	38	39	44	1.1	0.19	0.29	311 (96)	132 (55)
rs217386	NPC1L1	A	T.M.T.	-	+	43	32	27	0.87	0.19	0.23	281 (102)	56 (13)
rs3850634	ANGPTL3	G	T.M.T.	-	-	30	26	22	0.85	0.19	0.77	240 (85)	30 (9)
rs11887534	ABCG5/G8	C	J.A.K.	-	-	7.7	9.1	6.2	0.68	0.19	0.22	90 (29)	0 (0)
rs114067101	HLA-area	G	I.S.	+	+	5.8	3.3	5	1.5	0.2	0.83	13 (4)	0 (0)
rs72875462	ABCG8	A	I.S.	-	-	6.4	9	6.2	0.69	0.2	0.23	89 (29)	0 (0)
rs2920503	PPARG	T	I.S.	+	+	28	32	37	1.1	0.21	0.63	293 (104)	64 (27)
rs5882	CETP	G	J.A.K.	-	-	32	38	42	1.1	0.22	0.16	329 (124)	100 (36)
rs11136341	PLEC1	G	T.M.T.	+	+	41	37	41	1.1	0.22	0.27	316 (112)	107 (41)
rs1129555	GPAM	A	T.M.T.	+	-	31	33	29	0.88	0.22	0.32	280 (105)	57 (15)
rs117492019	ZNF274	T	I.S.	-	-	20	16	13	0.82	0.22	0.022	146 (50)	13 (4)
rs56130071	DNAH11	C	I.S.	+	+	19	21	24	1.2	0.23	0.62	231 (80)	39 (15)
rs719726	RSPO3	C	C.J.W.	+	-	48	44	40	0.9	0.23	0.43	297 (98)	124 (42)
rs12670798	DNAH11	C	T.M.T.	+	+	22	24	28	1.2	0.24	0.66	260 (89)	47 (20)
rs11164654	EVI5	T	I.S.	-	-	19	22	26	1.2	0.24	0.49	223 (75)	36 (22)
rs38855	MET	G	C.J.W.	-	+	48	46	42	0.91	0.24	0.79	305 (99)	148 (48)
rs3757354	MYLIP	T	T.M.T.	-	+	20	26	29	1.1	0.25	0.13	265 (94)	50 (21)
rs2030746	LOC84931	T	C.J.W.	+	-	41	35	39	1.1	0.26	0.86	309 (108)	92 (37)
rs340839	PROX1	A	I.S.	+	-	50	38	42	1.1	0.26	0.14	330 (117)	122 (40)
rs2230806	ABCA1	T	J.A.K.	+	-	27	22	19	0.86	0.26	0.28	190 (77)	18 (6)

rs4299376	ABCG5/8	G	T.M.T.	+	+	33	21	24	1.1	0.27	0.0094	235 (94)	27 (10)
rs1495743	NAT2	G	T.M.T.	+	+	24	25	22	0.88	0.27	0.12	257 (89)	33 (7)
rs10761731	JMJD1C	T	T.M.T.	+	-	45	38	34	0.9	0.28	0.18	321 (116)	91 (22)
rs77542162	ABCA6	G	I.S.	+	-	1.7	0.54	0.21	0.39	0.28		11 (1)	0 (0)
rs138570705	FRMD5	A	I.S.	-	-	3.3	1.8	2.8	1.6	0.29	0.77	28 (13)	0 (0)
rs2954029	TRIB1	T	T.M.T.	-	-	45	46	43	0.92	0.29	0.64	327 (119)	129 (40)
rs1121980	FTO	A	C.J.W.	+	+	45	43	39	0.91	0.29	0.096	326 (107)	87 (38)
rs731839	PEPD	G	C.J.W.	-	+	33	35	31	0.89	0.29	0.95	298 (103)	64 (21)
rs7759633	LPA	A	I.S.	+	-	14	9.3	12	1.3	0.3	0.74	130 (51)	7 (2)
rs9638182	MLXIPL	G	I.S.	+	-	19	17	15	0.85	0.3	0.69	176 (50)	32 (9)
rs186696265	LPA	T	I.S.	+	-	1.1	1.6	2.5	1.6	0.31	0.67	29 (7)	1 (0)
rs3816873	MTTP	C	J.A.K.	-	+	26	19	22	1.2	0.31	0.67	198 (79)	25 (12)
rs4719841	MIR148A	G	C.J.W.	-	-	40	37	40	1.1	0.31	0.84	285 (97)	101 (45)
rs1358980	VEGFA	T	I.S.	+	+	49	47	50	1.1	0.32	0.052	348 (128)	144 (53)
rs405509	APOE	T	J.A.K.	+	-	50	45	42	0.93	0.32	0.53	326 (115)	117 (38)
rs268	LPL	G	J.A.K.	+	+	2	2.2	3.2	1.5	0.33	0.9	37 (15)	0 (0)
rs4253776	PPARA	G	C.J.W.	+	+	12	7.9	6	0.76	0.33	0.86	81 (28)	1 (0)
rs3177928	HLA	A	T.M.T.	+	+	16	21	24	1.1	0.34	0.84	240 (89)	31 (11)
rs7811265	MLXIPL	G	T.M.T.	+	-	19	17	15	0.86	0.34	0.73	175 (50)	33 (9)
rs10195252	COBLL1	C	T.M.T.	-	-	46	34	38	1.1	0.36	0.54	298 (122)	80 (27)
rs1553318	TIMD4	G	T.M.T., I.S.	-	-	36	32	35	1.1	0.36	0.38	291 (97)	86 (34)
rs17585887	CITED2	T	I.S.	+	+	40	50	46	0.93	0.36	0.74	309 (121)	169 (48)
rs8077889	MPP3	C	C.J.W.	+	+	26	21	23	1.1	0.38	0.77	222 (80)	42 (14)
rs645040	MSL2L1	G	T.M.T.	-	-	25	14	12	0.84	0.38	0.052	146 (52)	7 (2)
rs2954022	TRIB1	A	T.M.T.	-	-	45	47	44	0.94	0.4	0.7	331 (123)	134 (41)
rs909802	TOP1	C	T.M.T.	-	-	55	46	43	0.94	0.41	0.65	349 (129)	128 (36)
rs11613352	LRP1	T	T.M.T.	+	+	20	23	25	1.1	0.42	0.35	231 (78)	39 (20)
rs61352607	LRP1	T	I.S.	+	+	20	23	25	1.1	0.43	0.34	231 (78)	39 (20)

rs2068888	CYP26A1	A	T.M.T.	-	-	49	47	49	1.1	0.47	0.84	318 (109)	159 (61)
rs4722551	MIR148A	C	C.J.W.	+	-	18	23	21	0.91	0.48	0.83	232 (71)	41 (13)
rs11648003	HPR	G	I.S.	+	+	23	22	24	1.1	0.49	0.42	203 (83)	41 (15)
rs5763662	MTMR3	T	C.J.W.	+	-	2.4	3.1	2.4	0.75	0.49	0.12	30 (11)	1 (0)
rs1800562	HFE	A	T.M.T.	-	-	5.1	3.8	4.7	1.2	0.5	0.35	64 (22)	0 (0)
rs12027135	LDLRAP1	A	T.M.T.	-	-	47	46	49	1.1	0.5	0.23	328 (115)	142 (56)
rs1367117	APOB	A	T.M.T.	+	+	32	28	30	1.1	0.52	0.32	256 (95)	64 (22)
rs1169314	HNF1A	G	I.S.	+	+	33	31	33	1.1	0.52	0.061	284 (104)	59 (25)
rs7205804	CETP	A	T.M.T.	-	-	43	42	44	1.1	0.54	0.77	344 (120)	124 (44)
rs3890182	ABCA1	A	J.A.K.	-	-	12	11	9.4	0.89	0.55	0.23	114 (42)	5 (1)
rs193042029	TM4SF5	G	I.S.	-	-	0.37	3	2.4	0.78	0.55	0.93	37 (11)	1 (0)
rs2929282	FRMD5	T	T.M.T.	-	-	5.1	4.9	6	1.2	0.56	0.78	54 (22)	5 (3)
rs3843482	HMGCR	G	I.S.	+	+	38	44	42	0.95	0.56	0.24	342 (116)	111 (37)
rs2943645	IRS1	C	T.M.T.	+	-	37	37	39	1.1	0.57	0.55	297 (105)	101 (38)
rs2332328	NYNRIN	T	T.M.T.	+	-	51	38	40	1	0.6	0.24	316 (106)	99 (40)
rs267733	ANXA9-CERS2	G	C.J.W.	-	+	15	14	13	0.9	0.6	0.66	155 (58)	12 (1)
rs17404153	ACAD11	T	C.J.W.	-	+	12	18	16	0.92	0.61	0.3	195 (66)	19 (5)
rs1799816	INSR	T	J.A.K.	+	+	0.93	0.64	0.43	0.67	0.61		4 (2)	0 (0)
rs10822145	JMJD1C	T	I.S.	+	-	51	48	46	0.96	0.62	0.46	345 (131)	151 (42)
rs2412710	CAPN3	A	T.M.T.	-	-	3.5	0.26	0.43	1.7	0.63	0.38	5 (2)	0 (0)
rs3780181	VLDLR	G	C.J.W.	-	+	5.6	5.3	6	1.1	0.63	0.94	61 (24)	4 (2)
rs59379014	ST3GAL4	T	C.J.W.	+	-	6.3	8	7	0.88	0.63	0.55	88 (31)	1 (1)
rs3198697	PDXDC1	T	C.J.W.	+	-	42	41	42	1	0.64	0.28	308 (115)	118 (38)
rs183130	CETP	T	J.A.K.	-	-	30	28	29	1.1	0.65	0.36	274 (98)	58 (20)
rs247616	CETP	T	T.M.T.	-	-	30	28	29	1.1	0.65	0.36	274 (98)	58 (20)
rs247617	CETP	A	I.S.	-	-	30	28	29	1.1	0.65	0.37	274 (98)	58 (20)
rs821840	CETP	G	I.S.	-	-	29	28	29	1.1	0.65	0.37	274 (98)	58 (20)
rs4530754	CSNK1G3	A	C.J.W.	+	-	61	50	52	1	0.65	0.7	328 (122)	181 (52)
rs4307732	ST3GAL4	A	I.S.	+	-	9.4	16	15	0.93	0.65	0.48	176 (56)	14 (7)

rs112374545	LDLR	T	I.S.	-	-	12	11	9.8	0.92	0.65	0.64	114 (42)	5 (2)
rs6511720	LDLR	T	T.M.T.	-	-	11	11	9.8	0.92	0.65	0.64	115 (42)	5 (2)
rs6882076	TIMD4	T	T.M.T.	-	-	36	33	34	1	0.66	0.35	287 (95)	86 (33)
rs5756931	PLA2G6	C	T.M.T.	-	-	41	39	38	0.96	0.66	0.46	327 (112)	76 (32)
rs4665710	APOB	A	I.S.	-	-	22	26	27	1	0.69	0.98	271 (96)	32 (16)
rs419132	HLA-area	G	I.S.	+	+	30	16	15	0.94	0.69	0.55	174 (61)	16 (5)
rs2000999	HPR	A	T.M.T.	+	+	19	19	21	1.1	0.7	0.38	194 (72)	27 (12)
rs58542926	TM6SF2	T	I.S.	-	-	8.1	5.9	5.3	0.9	0.7	0.28	62 (25)	3 (0)
rs1042034	APOB	C	T.M.T.	-	-	21	26	27	1	0.71	0.96	273 (96)	33 (16)
rs79588679	GATA6	T	I.S.	-	+	16	17	16	0.94	0.71	0.54	174 (69)	14 (3)
rs34894639	MSL2L1,PCCB	T	I.S.	-	-	26	19	18	0.94	0.71	0.37	170 (61)	14 (6)
rs11591147	PCSK9	T	C.J.W., J.A.K.	-	-	3.5	4	3.4	0.86	0.71	0.93	49 (14)	1 (1)
rs174551	FADS1	C	I.S.	-	+	32	40	39	0.97	0.72	0.62	296 (96)	97 (36)
rs2540948	CEP68	C	I.S.	+	-	37	32	31	0.97	0.73	0.99	292 (107)	66 (18)
rs10401969	CILP2	C	T.M.T.	-	-	7.3	5.8	5.3	0.91	0.73	0.26	61 (25)	4 (0)
rs1801689	APOH-PRXCA	C	C.J.W.	+	-	3.3	0.85	1.1	1.3	0.74	0.83	16 (5)	0 (0)
rs649129	ABO	T	T.M.T.	+	-	22	20	19	0.96	0.74	0.92	132 (45)	13 (6)
rs73001065	CILP2	C	I.S.	-	-	6.4	5.8	5.3	0.92	0.74	0.27	61 (25)	3 (0)
rs12310367	ZNF664	G	T.M.T.	-	-	38	28	29	1	0.75	0.27	268 (92)	56 (21)
rs140244541	FAM117B	A	I.S.	-	-	16	13	12	0.94	0.75	0.86	129 (49)	10 (4)
rs11776767	PINX1	C	T.M.T.	-	-	41	31	30	0.97	0.76	0.74	286 (99)	61 (20)
rs3902941	MAFB	G	T.M.T.	-	-	31	26	25	0.97	0.77	0.82	267 (93)	35 (12)
rs550057	ABO	T	I.S.	+	+	28	31	30	0.97	0.78	0.94	269 (96)	53 (22)
rs11220462	ST3GAL4	A	T.M.T.	+	+	12	19	18	0.96	0.78	0.28	211 (70)	15 (8)
rs9297994	CYP7A1	G	I.S.	+	+	36	38	39	1	0.79	0.93	294 (104)	104 (39)
rs1169288	HNF1A	C	J.A.K., T.M.T.	+	+	35	39	40	1	0.8	0.09	317 (119)	84 (34)
rs586178	LDLRAP1	C	I.S.	+	+	45	47	46	0.98	0.8	0.58	265 (87)	125 (46)
rs12916	HMGCR	C	T.M.T.	+	+	40	46	45	0.98	0.8	0.29	353 (122)	122 (44)

rs2297374	LPA	T	C.J.W.	-	-	40	34	33	0.98	0.8	0.66	302 (93)	89 (30)
rs2255811	GPR85	G	I.S.	-	+	28	24	23	0.97	0.8	0.71	211 (78)	32 (14)
rs1250229	FN1	T	C.J.W.	-	-	25	20	21	1	0.81	0.35	243 (82)	23 (7)
rs174583	FADS1-2-3	T	T.M.T.	-	+	34	42	41	0.98	0.82	0.69	314 (98)	111 (47)
rs1030431	CYP7A1	A	T.M.T.	+	+	35	31	30	0.98	0.82	0.45	266 (89)	62 (24)
rs364585	SPTLC3	A	C.J.W.	-	+	37	34	33	0.98	0.83	0.58	315 (110)	64 (23)
rs442177	KLHL8	G	T.M.T.	-	-	40	48	49	1	0.84	0.56	332 (114)	157 (57)
rs1077835	LIPC	G	I.S.	+	+	22	27	27	1	0.85	0.72	257 (95)	44 (16)
rs514230	IRF2BP2	A	T.M.T.	-	-	49	43	44	1	0.87	0.61	326 (115)	147 (45)
rs174529	FADS1	C	I.S.	-	+	36	42	43	1	0.89	0.61	317 (100)	117 (50)
rs1321257	GALNT2	G	T.M.T.	+	+	39	43	43	0.99	0.89	0.51	329 (117)	132 (42)
rs769452	APOE	C	J.A.K.	+	+	0.36	0.58	0.64	1.1	0.9	0.46	6 (3)	0 (0)
rs2618568	SNX5	C	I.S.	+	+	44	37	38	1	0.9	0.15	319 (113)	88 (32)
rs9686661	MAP3K1	T	T.M.T.	+	+	20	14	15	1	0.91	0.18	150 (57)	8 (6)
rs10864728	GALNT2	A	I.S.	+	+	39	43	43	0.99	0.91	0.48	326 (116)	129 (42)
rs2807834	MOSC1	T	T.M.T.	-	-	30	29	29	1	0.92	0.86	276 (91)	69 (23)
rs8023503	LIPC	T	J.A.K.	-	+	1.4	2	1.9	0.95	0.92	0.89	22 (8)	0 (0)
rs11153594	FRK	T	T.M.T.	-	-	41	40	41	1	0.94	0.47	319 (121)	108 (34)
rs1832007	AKR1C4	G	C.J.W.	-	-	12	13	13	1	0.95	0.47	142 (47)	17 (7)
rs261342	LIPC	G	T.M.T.	+	+	22	23	23	0.99	0.96	0.83	222 (80)	39 (13)
rs10490626	INSIG2	A	C.J.W.	-	-	8.2	6.7	6.6	0.99	0.96	0.8	78 (27)	3 (2)
rs11065987	BRAP	G	T.M.T.	-	+	44	38	38	1	0.97	0.71	299 (106)	94 (35)
rs2035403	KLHL8	G	I.S.	+	+	38	40	40	1	0.98	0.94	318 (110)	108 (37)
rs11621792	NYNRIN	T	I.S.	+	-	49	36	36	1	0.98	0.22	267 (91)	79 (33)
rs12594375	LIPC	A	J.A.K.	-	+	1.2	1.9	1.9	1	0.99	0.91	25 (9)	0 (0)
rs2247056	HLA	T	T.M.T.	-	-	21	23	23	1	0.99	0.59	239 (71)	41 (19)
rs174546	FADS1-2-3	T	T.M.T.	-	+	32	42	42	1	0.99	0.54	317 (99)	113 (48)
