# Supplementary material for: The Contribution of GWAS Loci in Familial Dyslipidemias
Source: PLoS Genet. 2016 May 26;12(5):e1006078. doi: 10.1371/journal.pgen.1006078 (PMC4882070; doi:10.1371/journal.pgen.1006078)
Supplement: S4 Table — In the EUFAM study, dyslipidemia was established based on levels of total cholesterol, triglycerides, or both that were ≥ 90th Finnish age- and sex-specific population percentile. The population percentiles were derived from FINMONICA, a large population survey performed in 1992. The percentile estimates and a description of the polynomial regression analyses used to establish them have been reported in detail previously [21]. (PDF) [file pgen.1006078.s010.pdf]

Age (years)	Total cholesterol		Triglycerides	
	Males	Females	Males	Females
–30	6.2	6.1	2.4	1.6
30–35	6.6	6.2	2.7	1.7
35–40	7.0	6.4	2.9	1.8
40–45	7.2	6.6	3.2	1.9
45–50	7.4	6.8	3.4	2.0
50–55	7.5	7.1	3.5	2.1
55–60	7.5	7.3	3.5	2.3
60–	7.4	7.6	3.5	2.5
